# HIV-1 Tat immunization restores immune homeostasis and attacks the HAART-resistant blood HIV DNA: results of a randomized phase II exploratory clinical trial

**DOI:** 10.1186/s12977-015-0151-y

**Published:** 2015-04-29

**Authors:** Fabrizio Ensoli, Aurelio Cafaro, Anna Casabianca, Antonella Tripiciano, Stefania Bellino, Olimpia Longo, Vittorio Francavilla, Orietta Picconi, Cecilia Sgadari, Sonia Moretti, Maria R Pavone Cossut, Angela Arancio, Chiara Orlandi, Leonardo Sernicola, Maria T Maggiorella, Giovanni Paniccia, Cristina Mussini, Adriano Lazzarin, Laura Sighinolfi, Guido Palamara, Andrea Gori, Gioacchino Angarano, Massimo Di Pietro, Massimo Galli, Vito S Mercurio, Francesco Castelli, Giovanni Di Perri, Paolo Monini, Mauro Magnani, Enrico Garaci, Barbara Ensoli

**Affiliations:** Pathology and Microbiology, San Gallicano Institute, Istituti Fisioterapici Ospitalieri, Rome, Italy; National AIDS Center, Istituto Superiore di Sanità, Viale Regina Elena 299, Rome, 00161 Italy; Department of Biomolecular Science, University of Urbino, Urbino, Italy; Division of Infectious Diseases, University Policlinic of Modena, Modena, Italy; Division of Infectious Diseases, S. Raffaele Hospital, Milan, Italy; Unit of Infectious Diseases, University Hospital of Ferrara, Ferrara, Italy; Department of Infectious Dermatology, San Gallicano Hospital, Rome, Italy; Division of Infectious Diseases, San Gerardo Hospital, Monza, Italy; Division of Infectious Diseases, University of Bari, Policlinic Hospital, Bari, Italy; Unit of Infectious Diseases, S.M. Annunziata Hospital, Florence, Italy; Institute of Tropical and Infectious Diseases, L. Sacco Hospital, University of Milan, Milan, Italy; Department of Infectious Diseases, S. Maria Goretti Hospital, Latina, Italy; Division of Tropical and Infectious Diseases, Spedali Civili, Brescia, Italy; Clinic of Infectious Diseases, Amedeo di Savoia Hospital, Turin, Italy; Istituto Superiore di Sanità, Rome, Italy, present address University of Tor Vergata, Rome, 00173 Italy

**Keywords:** HIV-1, Tat protein, Vaccine, HAART, Antibodies, Neutralization, Proviral DNA, CD38^+^HLA-DR^+^/CD8^+^ T cells

## Abstract

**Background:**

The phase II multicenter, randomized, open label, therapeutic trial (ISS T-002, Clinicaltrials.gov NCT00751595) was aimed at evaluating the immunogenicity and the safety of the biologically active HIV-1 Tat protein administered at 7.5 or 30 μg, given 3 or 5 times monthly, and at exploring immunological and virological disease biomarkers. The study duration was 48 weeks, however, vaccinees were followed until the last enrolled subject reached the 48 weeks.

Reported are final data up to 144 weeks of follow-up. The ISS T-002 trial was conducted in 11 clinical centers in Italy on 168 HIV positive subjects under Highly Active Antiretroviral Therapy (HAART), anti-Tat Antibody (Ab) negative at baseline, with plasma viremia <50 copies/mL in the last 6 months prior to enrollment, and CD4^+^ T-cell number ≥200 cells/μL. Subjects from a parallel observational study (ISS OBS T-002, Clinicaltrials.gov NCT0102455) enrolled at the same clinical sites with the same criteria constituted an external reference group to explore biomarkers of disease.

**Results:**

The vaccine was safe and well tolerated and induced anti-Tat Abs in most patients (79%), with the highest frequency and durability in the Tat 30 μg groups (89%) particularly when given 3 times (92%). Vaccination promoted a durable and significant restoration of T, B, natural killer (NK) cells, and CD4^+^ and CD8^+^ central memory subsets. Moreover, a significant reduction of blood proviral DNA was seen after week 72, particularly under PI-based regimens and with Tat 30 μg given 3 times (30 μg, 3x), reaching a predicted 70% decay after 3 years from vaccination with a half-life of 88 weeks. This decay was significantly associated with anti-Tat IgM and IgG Abs and neutralization of Tat-mediated entry of oligomeric Env in dendritic cells, which predicted HIV-1 DNA decay. Finally, the 30 μg, 3x group was the only one showing significant increases of NK cells and CD38^+^HLA-DR^+^/CD8^+^ T cells, a phenotype associated with increased killing activity in elite controllers.

**Conclusions:**

Anti-Tat immune responses are needed to restore immune homeostasis and effective anti-viral responses capable of attacking the virus reservoir. Thus, Tat immunization represents a promising pathogenesis-driven intervention to intensify HAART efficacy.

**Electronic supplementary material:**

The online version of this article (doi:10.1186/s12977-015-0151-y) contains supplementary material, which is available to authorized users.

## Background

HIV-1 persistence in reservoirs is associated with immune activation and T and B cell dysfunction, which represent the unmet needs of HAART [1-5], and are the cause of increased patients’ morbidity and mortality due to non-AIDS related diseases [1,3]. In fact, residual virus replication and cell-to-cell virus transmission still occur under a suppressive HAART and sustain the replenishment of HIV-1 reservoirs [5-7]. As a consequence, no reduction of blood HIV-1 DNA is observed after a few years of suppressive HAART, although levels appear to be lower in Protease Inhibitors (PI)- than in Non-Nucleoside Reverse Transcriptase Inhibitors (NNRTI)-treated patients [8]. This HAART-resistant virus reservoir and associated disorders represent a major problem in the clinical management of HIV-infected patients and a serious economical burden for the National Health Systems.

Intervention strategies aimed at attacking the virus reservoir and intensifying HAART efficacy have failed to date [9-12]. However, a “pathogenesis-driven” approach targeting viral products responsible of immune dysregulation, virus transmission and maintenance of virus reservoirs should be able to intensify HAART.

Tat is a key HIV virulence factor, which plays pivotal roles in virus gene expression, replication, transmission and disease progression [13-20]. Tat is produced very early upon infection [13-15] and continue to be expressed under HAART [21-23], is released extracellularly, accumulates in tissues, and exerts effects on both the virus and the immune system [15-25] that make it an optimal candidate for therapeutic immunization and HAART intensification [22,26-28].

Extracellular Tat induces HIV co-receptor expression [29], activates virus replication and efficiently increases virus transmission to neighbor cells [15,23,30-32]. Further, extracellular Tat, which has been shown to be present on highly purified virions [33], binds Env spikes present on virus particles forming a virus entry complex that favors infection of dendritic cells (DC) and transmission to T cells, key components of the virus reservoir [32]. This occurs by redirecting virus entry from the canonical receptors to RGD-binding integrins (α5β1, αvβ3, αvβ5) that Tat uses as receptors to enter DC [32]. Of note, by binding the Env C-C chemokine receptor type 5 co-receptor binding sites, Tat shields Env from anti-HIV Abs, thus inhibiting virus neutralization, which, however, can be restored and further increased by anti-Tat Abs [32]. Accordingly, mucosal immunization of monkeys with combined Tat and oligomeric Env protected animals from intrarectal virus challenge, blocking virus spread from the rectum to local lymph nodes [32]. Thus, Tat-specific Abs appear to be key to prevent HIV acquisition and spreading. Indeed, Tat vaccination in nonhuman primates can prevent or control infection with pathogenic SHIV [34,35], as recently confirmed by others, and found to correlate with anti-Tat Abs [36]. Notably, anti-Tat Abs are uncommon in natural infection [37] and, when present, correlate with the asymptomatic state, higher CD4^+^ T cell number and lower viral load, and with reduced or absent disease progression, particularly in infection with B clade viruses and when antibodies are persistent and characterized by multiple isotypes and high titers [38-41].

Moreover, Tat alters the homeostatic regulation of the immune system promoting an excessive and improper immune stimulation that prepares target cells for virus propagation while disabling an effective immune control. For example, extracellular Tat induces DC maturation [24,42] toward a Th1-type immune response, activates T cells independently from the engagement of the T-cell receptor [23,30], activates the immunoproteasome leading to alterations of cytotoxic T cell epitope presentation in CD8^+^ T cells [25,43], while skewing the response towards an “effector” and “effector memory” phenotype [44,45], which is accompanied by a loss of B cells [45]. CD8^+^ T cell hyperactivation is associated with functional deficit and is followed by effector cell apoptosis [46-49]. Notably, all these alterations are hallmarks of the chronic immune dysregulation observed in HIV-infected patients, which is only partially reverted by HAART [46-48].

Thus, the immune dysregulation induced by Tat appears to be pivotal in the establishment and maintenance of HIV infection, since it increases target cell recruitment, permissivity, virus reactivation and transmission, while disarming the host immune defense. This suggested that targeting Tat represents a pathogenesis-driven intervention to intensify HAART efficacy.

After the successful completion of randomized, placebo controlled, phase I trials with the biologically active HIV-1 Tat protein [26-28], a open-label randomized phase II trial with Tat was conducted in 168 anti-Tat Ab-negative, virologically suppressed HAART-treated subjects (ISS T-002, ClinicalTrials.gov NCT00751595). Seventy-nine subjects of the observational study (ISS OBS T-002, Clinicaltrials.gov NCT0102455) conducted in parallel at the same centers served as an external reference group for immunological and virological disease biomarkers, according to regulatory requirements [50].

Results of an exploratory interim analysis [22], up to 48 weeks, on 87 trial subjects which completed the treatment phase indicated earlier that Tat immunization not only was safe and immunogenic, but had also a positive impact on immune activation and T and B cell dysregulation that persist under effective antiretroviral treatment.

Here we report the results of immunogenicity and safety (primary and secondary endpoints of the trial, respectively) and of the immunological and virological disease biomarkers (second-line exploratory testing) in 168 individuals after trial completion (48 weeks), as well as after a follow-up of 144 weeks for a subgroup of vaccinees.

## Results

### Participants and treatment

**Figure 1:**
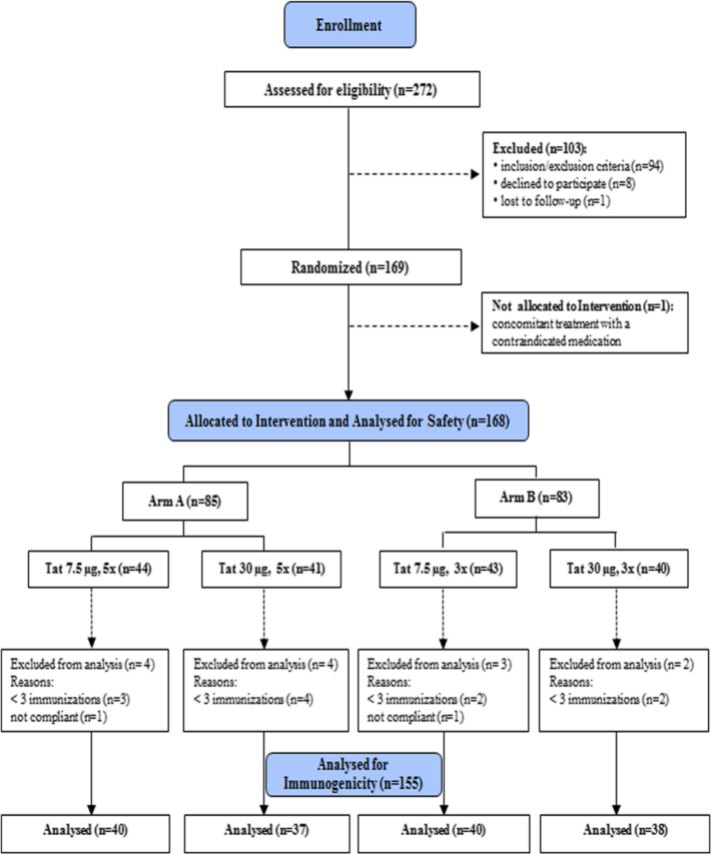
**Study flow chart.** Two hundred seventy-two HAART-treated patients were assessed for eligibility. One hundred three patients were excluded from the study (n = 94 for either not meeting the inclusion criteria or for meeting exclusion criteria; n = 8 declined to participate, and 1 was lost to follow-up). Thus, 169 subjects were randomized to one of the 4 treatment groups. One subject was excluded after randomization for concomitant treatment with a contraindicated medication, therefore 168 volunteers were allocated to intervention and analyzed for safety (safety population). Since 11 subjects (from the different treatment groups) received less than 3 immunizations and 2 subjects were non compliant with antiretroviral therapy, 155 subjects were considered for the analyses of immunogenicity (immunogenicity population).

Two hundred seventy-two volunteers were assessed for eligibility and 103 were excluded from the enrollment: 94 volunteers either did not meet inclusion criteria or met exclusion criteria, 8 declined to participate and 1 individual was lost to follow up (Figure 1).

One hundred sixty-eight HIV-1 infected HAART-treated, anti-Tat Ab negative adults were allocated to intervention and analyzed for safety. Since 11 subjects received less than 3 immunizations and 2 individuals were not therapy compliant, the immunogenicity population consisted of 155 patients randomized to receive the biologically active HIV-1 Tat protein at 7.5 μg or 30 μg, given 3 (3x) or 5 (5x) times at monthly intervals, respectively (Figure 1).

Seventy-nine anti-Tat Ab negative HAART-treated subjects enrolled with the same criteria at the same sites in the parallel observational study ISS OBS T-002 and analyzed simultaneously by the same core lab represented the external control group for disease biomarkers [50]. Baseline demographic and clinical characteristics of ISS T-002 and ISS OBS T-002 participants were comparable, although significant differences were detected for CD4 nadir and in the percentage of CD4^+^ T cells (Table 1). Since the trial protocol was amended during the study to include more immune compromised subjects (see *Methods*), both the pre- and post-amendment trial populations were also compared with OBS subjects (Table 2). The ISS T-002 study population after the protocol amendment did not show any significant difference as compared to the OBS group. No differences were observed at baseline in the demographic and clinical characteristics of the ISS T-002 study participants according to treatment groups (Table 3). Values of immunological and virological biomarkers at study entry for ISS T-002 and ISS OBS T-002 participants were comparable and no significant differences were detected except for B cell numbers (Table 4). Lower but still significant differences as compared to OBS subjects were still observed for B cells in trial subjects after the protocol amendment (Table 5). Nevertheless, in all cases B cell counts at baseline were well within the normal range in both T-002 and OBS subjects. No significant differences between trial subjects both pre- and post-amendment and OBS subjects were detected for the other immunological and virological parameters at baseline (Table 5).

**Table 1:** **Baseline characteristics of the ISS T-002 and ISS OBS T-002 study participants**

	**ISS T-002**	**ISS OBS T-002**	**p-value** ^**d**^
	**(** ***n*** **= 155)**	**(** ***n*** **= 79)**	
**Age**			
Mean ± s.d.^a^	41 ± 7	42 ± 6	0.6323
Range	24-55	24-55	
**Gender**			
Male	83%	75%	0.1200
Female	17%	25%	
**CD4** ^**+**^ **nadir (cells/μl)**			
Mean ± s.d.	308 ± 133	219 ± 140	<0.0001
Range	14-846	2-612	
**CD4** ^**+**^ **(cells/μl)**			
Mean ± s.d.	639 ± 220	657 ± 271	0.5450
Range	212-1490	208-1527	
**CD4** ^**+**^ **(%)**			
Mean ± s.d.	33 ± 8	31 ± 8	0.0257
Range	14-61	12-52	
**HIV RNA (copies/ml)**	<50^b^	<50^c^	
**Years from diagnosis of HIV**			
Mean ± s.d.	9 ± 6	11 ± 7	0.1789
Range	1-25	1-24	
**Years from HAART initiation**			
Mean ± s.d.	6 ± 4	6 ± 5	0.4984
Range	0-19	0-19	
**Current HAART regimen**			
NNRTI-based or NRTI	68%	62%	0.3305
PI-based	32%	38%	

*n* indicates the number of evaluable individuals; ^a^Standard deviation; ^b^Five subjects had values >50 copies/ml (between 58 and 91); ^c^Four subjects had values >50 copies/ml (between 64 and 118);

^d^ *t*-test was applied for continuous variables, Chi-square test was applied for categorical variables.

**Table 2:** **Baseline characteristics of ISS T-002 subjects pre- and post-protocol amendment and ISS OBS T-002 subjects**

	**ISS T-002 pre-amendment**	**ISS T-002 post-amendment**	**ISS OBS T-002**	**OBS vs pre-amendment T-002**	**OBS vs post-amendment T-002**
	**(** ***n*** **= 87)**	**(** ***n*** **= 81)**	**(** ***n*** **= 79)**	**p-value** ^**b**^	**p-value** ^**b**^
**Age**					
Mean ± s.d.^a^	41 ± 7	42 ± 7	42 ± 6	0.2963	0.8043
Range	26-54	23-55	24-55		
**Gender**					
Male	78%	86%	75%	0.5977	0.0604
Female	22%	14%	25%		
**CD4** ^**+**^ **nadir (cells/μl)**					
Mean ± s.d.	352 ± 114	251 ± 131	219 ± 140	<0.0001	0.1131
Range	252-846	14-585	2-612		
**CD4** ^**+**^ **(cells/μl)**					
Mean ± s.d.	693 ± 210	585 ± 215	657 ± 271	0.4018	0.0668
Range	425-1490	144-1250	208-1527		
**CD4** ^**+**^ **(%)**					
Mean ± s.d.	34 ± 7	32 ± 8	31 ± 8	0.0033	0.2596
Range	19-53	14-61	12-52		
**Years from diagnosis of HIV**					
Mean ± s.d.	9 ± 6	9 ± 7	11 ± 7	0.1583	0.3703
Range	1-23	1-25	1-24		
**Years from HAART initiation**					
Mean ± s.d.	6 ± 4	7 ± 5	6 ± 5	0.2552	0.9073
Range	0-19	0-19	0-19		
**Current HAART regimen**					
NNRTI-based or NRTI	75%	56%	62%	0.0784	0.3434
PI-based	25%	44%	38%		

*n* indicates the number of individuals; ^a^Standard deviation; ^b^ *t*-test was applied for continuous variables, Chi-square test was applied for categorical variables.

**Table 3:** **Baseline characteristics of the ISS T-002 study participants by treatment groups**

	**Tat 7.5 μg, 3x**	**Tat 7.5 μg, 5x**	**Tat 30 μg, 3x**	**Tat 30 μg, 5x**
	**(** ***n*** **= 40)**	**(** ***n*** **= 40)**	**(** ***n*** **= 38)**	**(** ***n*** **= 37)**
**Age**				
Mean ± s.d.^a^	40 ± 7	41 ± 6	43 ± 5	42 ± 8
Range	24-54	29-55	33-55	26-54
**Gender**				
Male	73%	90%	79%	92%
Female	27%	10%	21%	8%
**CD4** ^**+**^ **nadir (cells/μl)**				
Mean ± s.d.	287 ± 74	321 ± 139	337 ± 165	285 ± 136
Range	97-450	76-699	15-846	14-585
**CD4** ^**+**^ **(cells/μl)**				
Mean ± s.d.	660 ± 216	644 ± 233	617 ± 214	632 ± 222
Range	293-1490	214-1382	212-1362	286-1250
**CD4** ^**+**^ **(%)**				
Mean ± s.d.	34 ± 7	33 ± 7	33 ± 7	33 ± 10
Range	22-53	20-46	19-47	14-61
**Years from diagnosis of HIV**				
Mean ± s.d.	8 ± 6	10 ± 6	10 ± 7	9 ± 7
Range	1-21	2-25	1-23	1-24
**Years from HAART initiation**				
Mean ± s.d.	5 ± 4	6 ± 4	7 ± 5	7 ± 5
Range	0-19	0-19	0-18	1-17
**Current HAART regimen**				
NNRTI-based or NRTI	58%	75%	66%	76%
PI-based	42%	25%	34%	24%

*n* indicates the number of evaluable individuals; ^a^Standard deviation.

No significant differences were detected among treatment groups at baseline.

**Table 4:** **Immunological and virological parameters at baseline in ISS T-002 and ISS OBS T-002 study participants**

	**ISS T-002**		**ISS OBS T-002**		**p-value** ^**b**^
	***n***	**Mean ± s.d.** ^**a**^	***n***	**Mean ± s.d.** ^**a**^	
**Phenotype (cells/μL)**					
CD4^+^	152	661 ± 231	79	700 ± 300	0.2671
CD8^+^	152	802 ± 393	79	881 ± 360	0.1295
B	152	241 ± 121	79	307 ± 164	0.0006
NK	152	232 ± 152	79	268 ± 193	0.1231
**CD4** ^**+**^ **subsets (%)**					
Naïve	94	31 ± 14	31	28 ± 13	0.7057
Effector memory	94	22 ± 12	31	20 ± 8	0.5304
Central memory	94	47 ± 10	31	51 ± 9	0.2810
**CD8** ^**+**^ **subsets (%)**					
Naïve	94	26 ± 11	31	30 ± 11	0.0835
Effector memory	94	52 ± 13	31	47 ± 13	0.0756
Central memory	94	22 ± 10	31	23 ± 9	0.7302
**HIV DNA (log**_**10**_ **copies/10** ^**6**^ **CD4** ^**+**^ **cells)**	142	3.1 ± 0.5	54	3.0 ± 0.5	0.4381
Tat 7.5 μg, 3x	36	3.1 ± 0.6			
Tat 7.5 μg, 5x	36	3.0 ± 0.6			
Tat 30 μg, 3x	36	3.2 ± 0.5			
Tat 30 μg, 5x	34	3.0 ± 0.5			

"*n* indicates the number of individuals evaluable for each parameter according to residual specimen availability, since they were part of second line laboratory testing".

^a^Standard deviation; ^b^ *t*-test was applied.

**Table 5:** **Baseline Immunological-virological parameters in ISS T-002 subjects pre- and post-protocol amendment and OBS subjects**

	**ISS T-002 pre-amendment**		**ISS T-002 post-amendment**		**ISS OBS T-002**		**OBS vs pre-amendment T-002**	**OBS vs post-amendment T-002**
	***n***	**Mean ± s.d.** ^**a**^	***n***	**Mean ± s.d.**	***n***	**Mean ± s.d.**	**p-value** ^**b**^	**p-value** ^**b**^
**Phenotype (cells/μL)**								
CD4^+^	80	694 ± 236	72	624 ± 223	79	700 ± 300	0.8880	0.0755
CD8^+^	80	822 ± 344	72	780 ± 443	79	881 ± 360	0.2955	0.1299
B	80	232 ± 111	72	251 ± 132	79	307 ± 164	0.0009	0.0244
NK	80	237 ± 149	72	226 ± 159	79	268 ± 193	0.2596	0.1480
**CD4** ^**+**^ **subsets (%)**								
Naïve	21	32 ± 19	73	31 ± 12	31	28 ± 13	0.6337	0.7940
Effectors memory	21	23 ± 14	73	22 ± 12	31	20 ± 8	0.4377	0.6029
Central memory	21	45 ± 15	73	48 ± 9	31	51 ± 9	0.2314	0.3422
**CD8** ^**+**^ **subsets (%)**								
Naïve	21	26 ± 11	73	26 ± 12	31	30 ± 11	0.2004	0.0912
Effectors memory	21	53 ± 12	73	52 ± 14	31	47 ± 13	0.0683	0.1174
Central memory	21	20 ± 11	73	22 ± 10	31	23 ± 9	0.4262	0.9136
**HIV DNA (log**_**10**_ **copies/10** ^**6**^ **CD4** ^**+**^ **cells**	78	3.1 ± 0.5	64	3.1 ± 0.5	54	33.0 ± 0.5	0.4505	0.5276

"*n* indicates the number of individuals evaluable for each parameter according to residual specimen availability, since they were part of second line laboratory testing". ^a^Standard deviation; ^b^ *t*-test was applied.

### Immunogenicity

**Figure 2:**
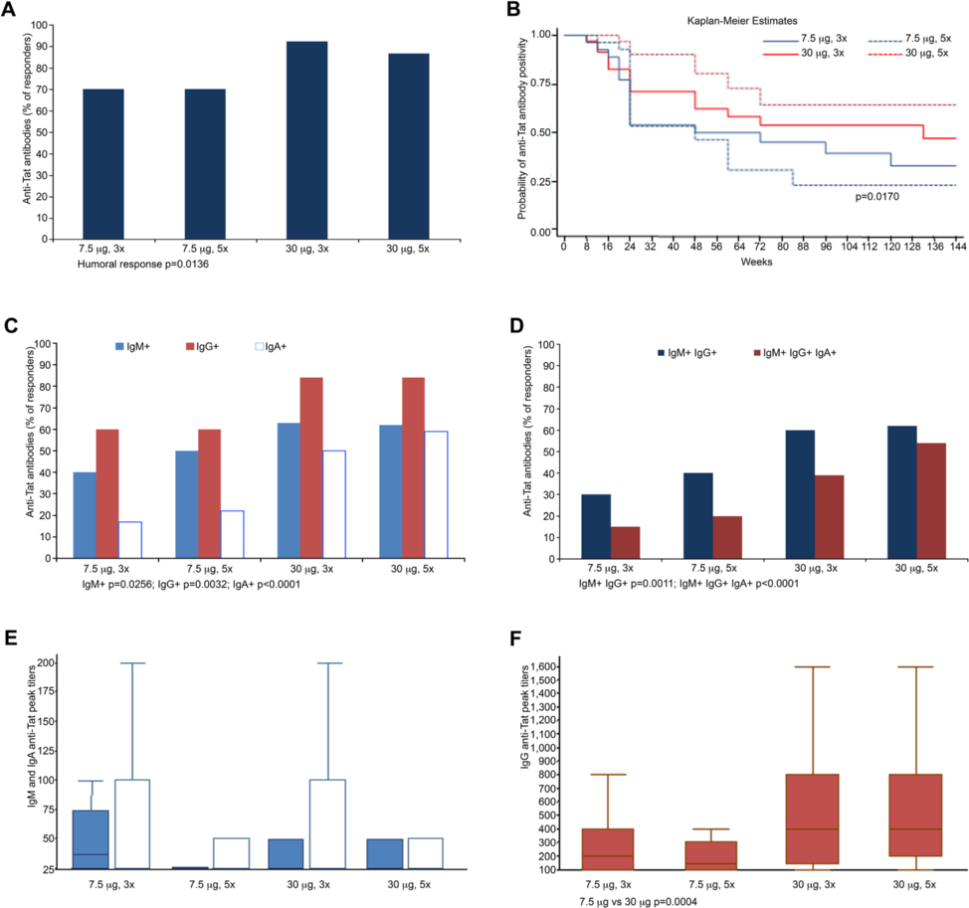
**Anti-Tat humoral immune response in vaccinees. (A)** Percentage of subjects producing Anti-Tat Abs (responders) after Tat immunization (7.5 μg, 3x n = 40; 7.5 μg, 5x n = 40; 30 μg, 3x n = 38; 30 μg, 5x n = 37). **(B)** Kaplan-Meier estimates showing the cumulative probability of anti-Tat Ab durability in responders stratified according to treatment groups and up to week 144 of follow-up (n = 123, median follow-up of 96 weeks). **(C)** Percentage of subjects in each treatment group producing anti-Tat IgM (light blue), IgG (red), or IgA (white) Abs. **(D)** Percentage of subjects in each treatment group producing two (IgM and IgG, in blue) or three (IgM, IgG and IgA, in red) anti-Tat Ab classes. **(E)** Anti-Tat IgM (light blue) and IgA (white) peak titers between 4 and 24 weeks since the first immunization in subjects positive for IgM (7.5 μg, 3x n = 16; 7.5 μg, 5x n = 20; 30 μg, 3x n = 24; 30 μg, 5x n = 23) or IgA anti-Tat Abs (7.5 μg, 3x n = 7; 7.5 μg, 5x n = 9; 30 μg, 3x n = 19; 30 μg, 5x n = 22). **(F)** Anti-Tat IgG Ab peak titers between 4 and 24 weeks since the first immunization in subjects positive for IgG anti-Tat Abs (7.5 μg, 3x n = 24; 7.5 μg, 5x n = 24; 30 μg, 3x n = 32; 30 μg, 5x n = 31). Box plots represent the median, 25^th^ and 75^th^ percentile, with the minimum and maximum values; the outliers are not represented in the graphs.

Tat immunization induced anti-Tat Abs in most vaccinees (79%), with the highest frequency in the Tat 30 μg groups (89%) and particularly after 3 administrations (92%) as compared to the 7.5 μg arms (70%) (Table 6 and Figure 2A). Statistically significant odds ratios were detected for Tat 30 μg 3x or 5x as compared to the reference (Tat 7.5 μg, 3x), whereas no differences were observed between 7.5 μg 5 x and the reference (Table 6).

**Table 6:** **ISS T-002: anti-Tat humoral and cellular immune response post-immunization**

	***n***	**Percentage (95% CI** ^**a**^ **)**	**Odds ratio** ^**b**^ **(95% CI** ^**a**^ **)**	**Odds ratio p-value**
**Anti-Tat humoral responses**				
Vaccinees	155	79 (73–86)		
Tat 7.5 μg, 3x	40	70 (56–84)	*reference*	
Tat 7.5 μg, 5x	40	70 (56–84)	1.0 (0.5; 2.2)	1.0000
Tat 30 μg, 3x	38	92 (84–100)	5.0 (1.8; 13.9)	0.0021
Tat 30 μg, 5x	37	86 (75–97)	2.7 (1.1; 6.7)	0.0272
**Anti-Tat cellular responses**				
Vaccinees	155	84 (79–90)		
Tat 7.5 μg, 3x	40	80 (68–92)	*reference*	
Tat 7.5 μg, 5x	40	88 (77–98)	1.7 (0.7; 4.4)	0.3253
Tat 30 μg, 3x	38	87 (76–98)	1.6 (0.6; 4.2)	0.2900
Tat 30 μg, 5x	37	84 (72–96)	1.3 (0.5; 3.2)	0.5758
*Comparison among groups*				0.6697^c^

*n* indicates the number of individuals evaluable for each parameter.

^a^Confidence interval; ^b^Logistic regression model; ^c^Mantel-Haenszel Chi-Square test.

**Figure 3:**
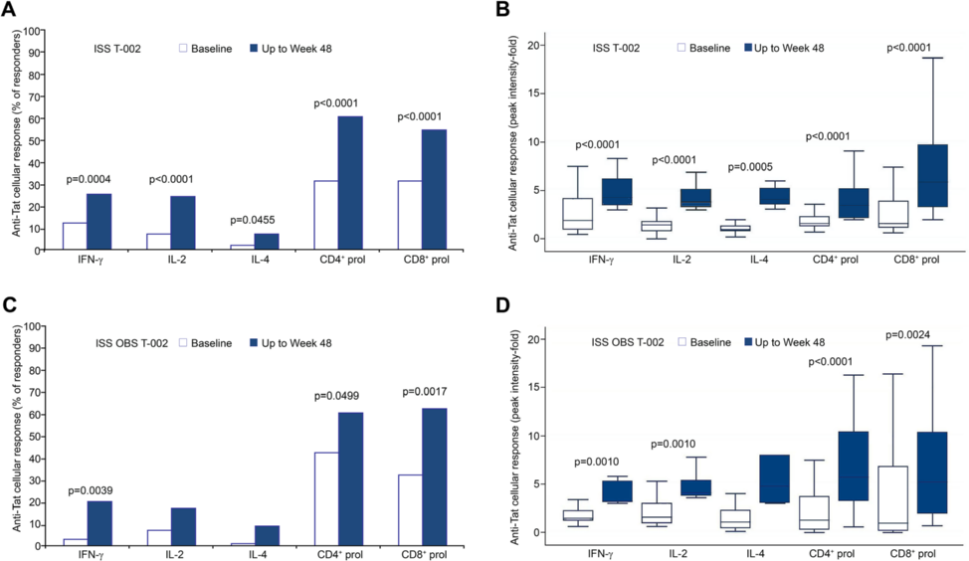
**Anti-Tat cellular immune response.** IFN-γ, IL-2 or IL-4 production and CD4^+^ or CD8^+^ T cell proliferation to Tat in **(A)** vaccinees or **(C)** OBS subjects. The percentages of responders at baseline (white bars) and up to 48 weeks from baseline (blue bars) (vaccinees: IFN-γ, IL-2, or IL-4 n = 151, proliferation n = 140; OBS: IFN-γ, IL-2, or IL-4 n = 60, proliferation n = 54) are shown. Peak intensity of anti-Tat cellular responses in **(B)** vaccinees or **(D)** OBS subjects at baseline (white bars) and up to 48 weeks (blue bars) in responders (subjects with at least one positive response after baseline) (vaccinees: IFN-γ n = 39, IL-2 n = 38, IL-4 n = 12, CD4^+^ proliferation n = 86, CD8^+^ proliferation n = 77; OBS: IFN-γ n = 12, IL-2 n = 11, IL-4 n = 6, CD4^+^ proliferation n = 33, CD8^+^ proliferation n = 34). Box plots represent the median, 25^th^ and 75^th^ percentile, with the minimum and maximum values; the outliers are not represented in the graphs. The McNemar’s and Wilcoxon signed-rank tests were applied. P-values assess the values of frequencies and intensity, respectively, up to 48 weeks after immunization versus baseline values.

In the Tat 30 μg groups Abs persisted significantly longer, as compared to the Tat 7.5 μg groups (Figure 2B). The 30 μg doses were also more effective at inducing anti-Tat Abs of different isotypes (Figure 2C, D), and peak IgG titers (Figure 2E, F). Tat immunization also increased the percentage of responders and intensity of anti-Tat cellular responses, including IFN-γ, IL-4, IL-2 production and CD4^+^-, CD8^+^-T cell proliferation, as compared to baseline (Table 6 and Figure 3A, B) without significant differences among the treatment groups. Cellular responses to Tat were also present in OBS subjects (Figure 3C, D).

### Safety

Immunization with Tat was safe and well tolerated without any notable dose-dependent relationship. The adverse events (AE) reported during the study indicated that the Tat vaccine may induce mild injection site reactions, and systemic effects, which were generally well tolerated. A total of 103 patients experienced at least one AE during the 48 weeks of the study and the mean number of AE reported per patient was 3.63 (range 1–23). These AE were mainly mild and unrelated to the study treatment (Table 7). General disorders and administration site reactions were the most frequently reported AE with a possible, probable or definite relationship to study treatment (Additional file 1: Table S1). Serious AE were recorded in 10 vaccinees. For 8 of them the events were judged “unrelated (n. 6) or unlikely related (n. 2)” to the study drug. The remaining 2 patients with serious AE manifested in one case “possibly related” peripheral neurological symptoms (dysarthria, motor aphasia, facial weakness, tongue paresthesia associated with retrosternal constriction) which resolved after 10 days; in the other case a “probably related” right Bell’s paralysis was diagnosed which resolved within 3 months. These events were evaluated by the Data Safety Monitoring Board (DSMB) overseeing the study and judged as manifestations occurring more frequently in HIV-infected patients and also relatively common after the administration of different commercial vaccines. After the 48 weeks of the study, no relevant AE were recorded. Three subjects experienced serious AE judged as “unrelated” to the study treatment that included 2 subjects with hepatic enzymes increase and *S. aureus* infection and abdominal pain, respectively, which were completely resolved within 4 months, and a third subject with ischemic hemianopsia which was resolved with sequelae.

**Table 7:** **Adverse events occurred up to week 48 in the ISS T-002 study**

	**Treatment groups**								
	**Tat 7.5 μg, 3x**		**Tat 7.5 μg, 5x**		**Tat 30 μg, 3x**		**Tat 30 μg, 5x**		
	**N**	**%**	**N**	**%**	**N**	**%**	**N**	**%**	**Total**
Number of subjects in the safety population	43		44		40		41		168
Number of subjects with at least one adverse event	27	62.8	26	59.1	26	65.0	25	61.0	103
Serious adverse events	2	7.14	2	7.1	3	11.1	3	11.1	10
Non-Serious Adverse Events	26	92.9	26	92.9	24	88.9	24	88.9	100
Adverse Events leading to withdrawal	1	1.3	2	2.5	2	2.6	2	2.9	7
Deaths	0		0		0		0		0
*Relationship with study medication*									
Certain	3	8.6	3	8.6	4	12.5	8	23.5	18
Probable	1	2.9	5	14.3	3	4.0	3	8.8	12
Possible	4	11.5	3	8.6	4	12.5	2	5.9	13
Unlikely	2	5.7	3	8.6	1	3.3	0	0.0	6
Unrelated	25	71.4	21	60.0	20	62.5	21	61.8	87
*Severity*									
Mild	26	76.5	25	67.6	22	71.0	21	63.6	94
Moderate	6	17.6	8	21.6	7	22.6	9	27.3	30
Severe	2	5.9	4	10.8	2	6.4	3	9.1	11

Based on the overall safety data the DSMB judged the Tat vaccine as safe and well tolerated.

### Immune restoration

**Figure 4:**
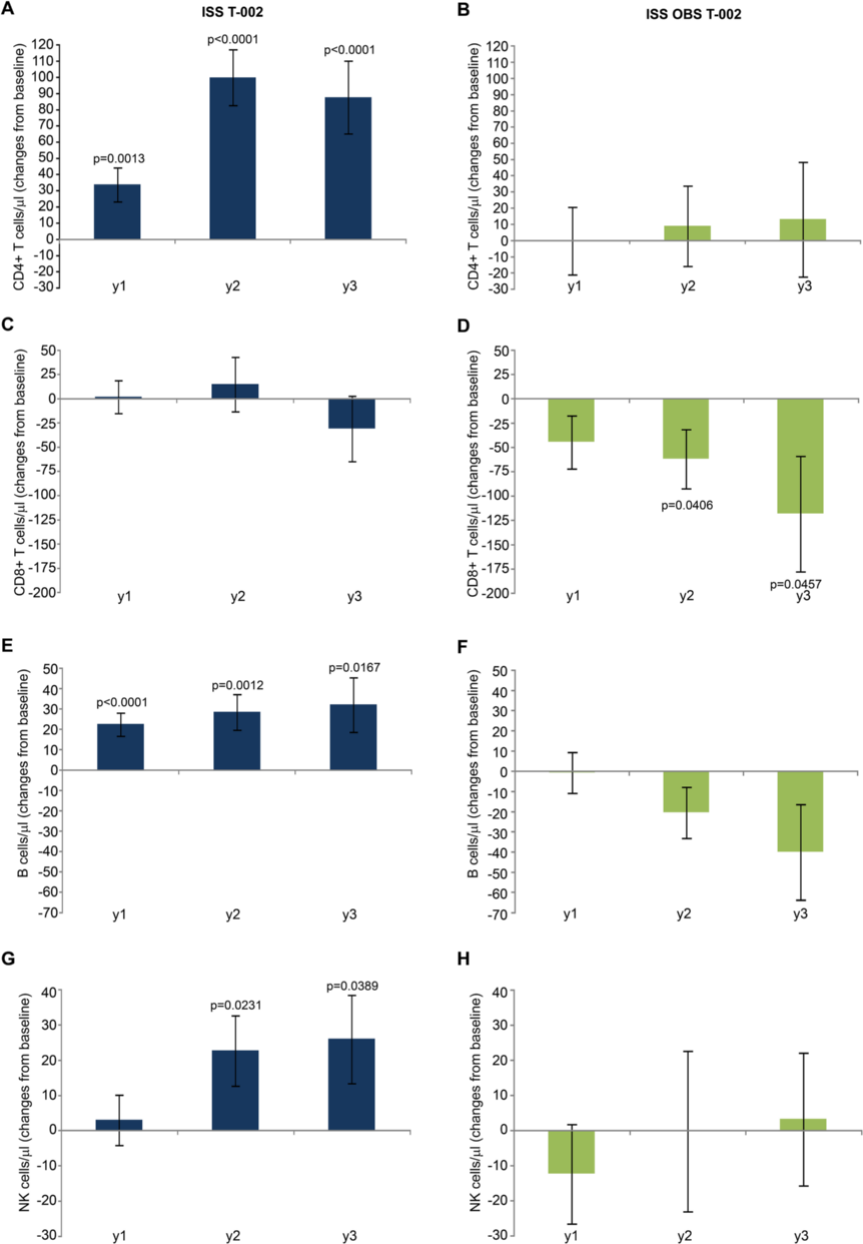
**CD4** ^**+**^ **, CD8** ^**+**^ **, B and NK cell numbers.** Changes from baseline of **(A, B)** CD4^+^, **(C, D)** CD8^+^, **(E, F)** B and **(G, H)** NK cells at years 1, 2 and 3 in vaccinees (left panels) (n = 152 at year 1 ; n = 114 at year 2 ; n = 69 at year 3) and in OBS subjects (right panels) (n = 79 at year 1 ; n = 42 at year 2 ; n = 30 at year 3), respectively. Data are presented as mean values with standard error. A longitudinal analysis for repeated measurements was applied. P-values assess the values at year 1, 2 or 3 after immunization versus baseline values.

A significant increase of CD4^+^ T cell number was observed early after Tat immunization, peaking at year 2 (up to about 100 cells/μl increase p < 0.0001) and persisting at year 3, while no significant changes from baseline were detected in OBS subjects during the 3 years of follow up (Figure 4A, B), as observed previously for successfully treated subjects after years of therapy (mean of 6 years for both ISS T-002 and OBS subjects) [51-53]. At the same time, Tat immunization maintained stable levels of CD8^+^ T cells, which, in contrast, progressively decreased in OBS subjects, particularly at year 2 and 3 (Figure 4C, D), as observed earlier during HAART [54-56]*.* An early and significant increase of B cells was also induced by Tat immunization that reached the highest value at year 3, whereas a trend to reduction was observed in OBS subjects (Figure 4E, F). Natural killer (NK) cell numbers were also significantly increased in vaccinees at year 2 and 3, while no significant changes were observed in OBS subjects (Figure 4 G, H). The pattern of B and NK cells seen in OBS is similar to what has been reported during HAART [57,58].

**Figure 5:**
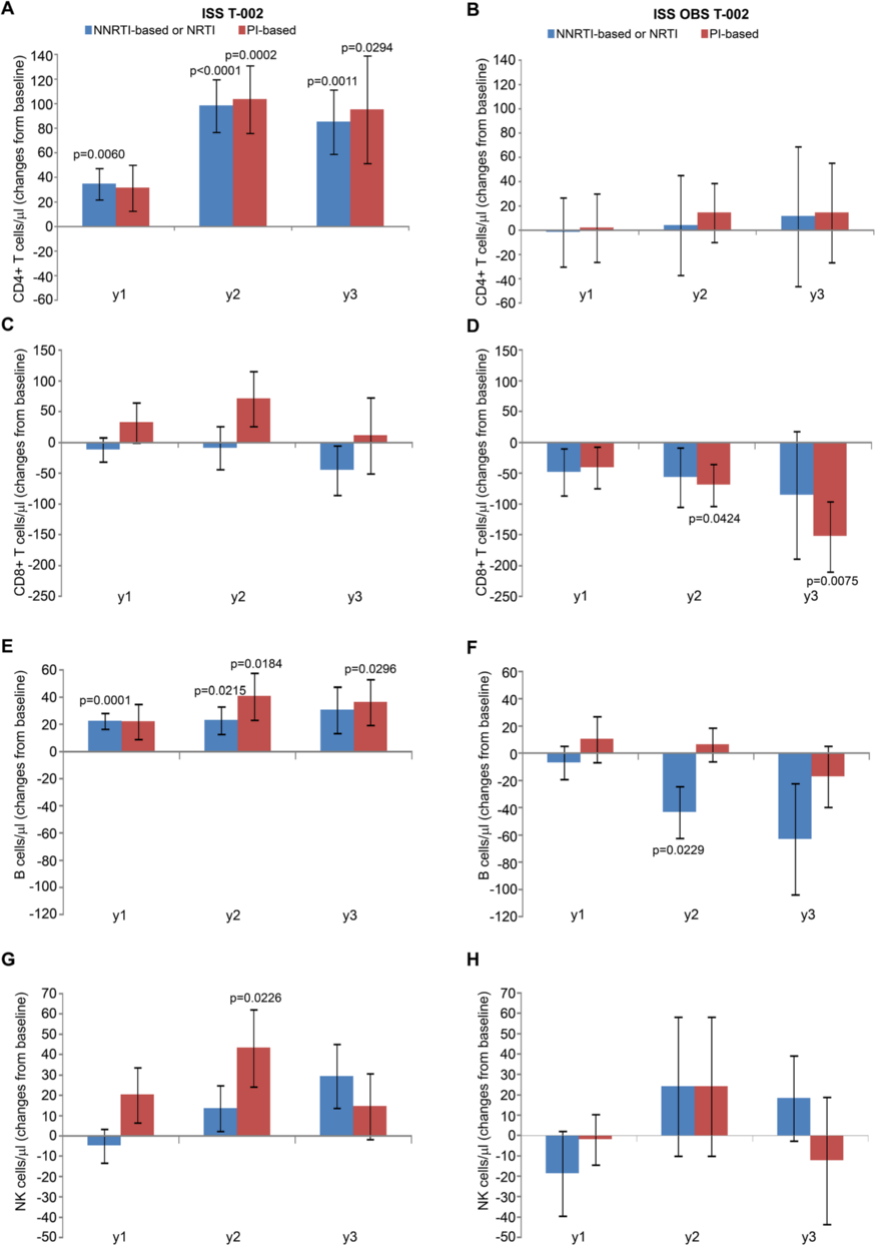
**CD4** ^**+**^ **, CD8** ^**+**^ **, B and NK cell numbers stratified by antiretroviral regimens.** Changes from baseline of **(A, B)** CD4^+^, **(C, D)** CD8^+^, **(E, F)** B and **(G, H)** NK cells at years 1, 2 and 3 according to antiretroviral regimens in vaccinees (left panels) (NNRTI- or NRTI-based n = 104 at year 1 ; n = 80 at year 2 ; n = 49 at year 3; PI-based n = 48 at year 1 ; n = 34 at year 2 ; n = 20 at year 3) and OBS subjects (right panels) (NNRTI- or NRTI-based n = 49 at year 1 ; n = 21 at year 2 ; n = 14 at year 3; PI-based n = 30 at year 1 ; n = 20 at year 2 ; n = 16 at year 3). Data are presented as mean values with standard error. A longitudinal analysis for repeated measurements was applied. P-values assess the values at year 1, 2 or 3 after immunization versus baseline values, both overall and stratified by drug regimens (NNRTI/NRTI- or PI-based).

In vaccinees, changes of CD4^+^ and CD8^+^ T cells, B and NK cells were comparable with both drug regimens (NNRTI/NRTI- or PI-based) (Figure 5). In contrast, the decreases of CD8^+^ T cells and B cells observed in OBS subjects were differently affected by the drug regimens. In particular, the CD8^+^ T cell loss was more profound, reaching statistical significance, under PI-based regimens (Figure 5D), whereas the B cell reduction occurred mostly under NNRTI/NRTI regimens (Figure 5F). Thus, Tat immunization during HAART restores T, B and NK cell numbers as compared to HAART alone.

**Figure 6:**
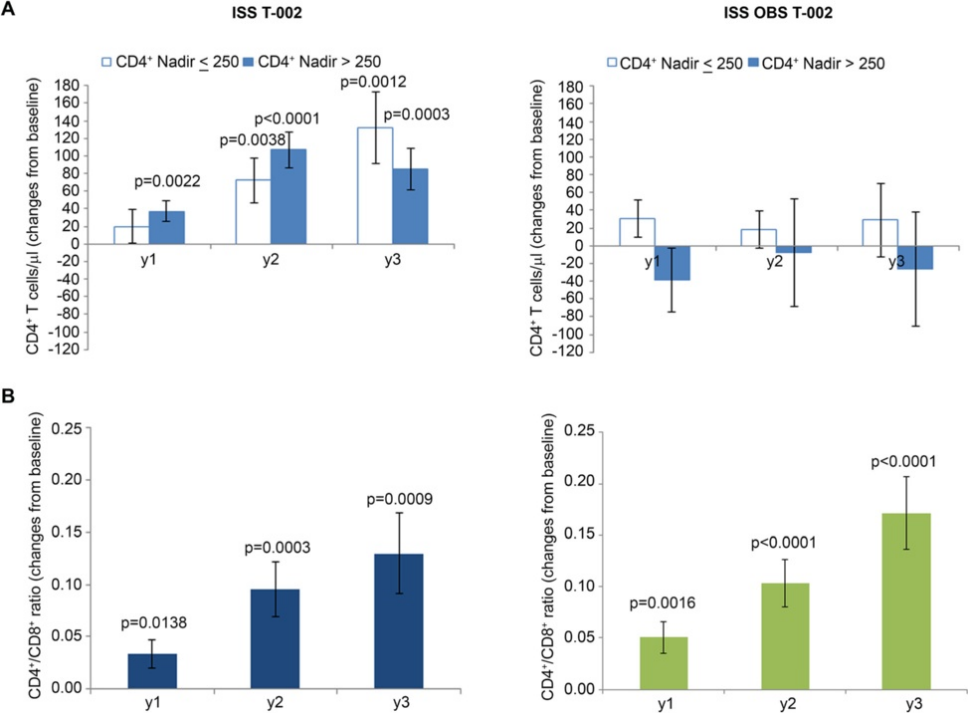
**CD4** ^**+**^ **T cells stratified by CD4** ^**+**^ **nadir and CD4** ^**+**^ **/CD8** ^**+**^ **T cell ratio.** Changes from baseline of **(A)** CD4^+^ by CD4^+^ nadir and **(B)** CD4^+^/CD8^+^ T cell ratio at years 1, 2 and 3 in vaccinees (left panels) (n = 152 at year 1 ; n = 114 at year 2 ; n = 69 at year 3) and in OBS subjects (right panels) (n = 79 at year 1 ; n = 42 at year 2 ; n = 30 at year 3), respectively. Vaccinees with CD4^+^ T cell nadir ≤250 cells/μl: n = 32, >250 cells/μl n = 120; OBS subjects with CD4+ T cell nadir ≤250 cells/μl: n = 45 ; >250 cells/μl n = 34. Data are presented as mean values with standard error. A longitudinal analysis for repeated measurements was applied. P-values assess the values at year 1, 2 or 3 after immunization versus baseline values.

Of note, the increase of CD4^+^ T cells after vaccination was independent from the nadir and at year 3 subjects with nadir ≤250 cells/μl had increases greater, although not significantly different, than those with a nadir >250 cells/μl (increase of 132 cells/μl p = 0.0012 vs 85/μl cells p = 0.0003, respectively) (Figure 6A).

The increase of CD4^+^ T cells and the maintenance of CD8^+^ T cells after vaccination were associated with a statistically significant increase of CD4^+^/CD8^+^ ratio overtime (Figure 6B). This was observed also in OBS subjects. However, in vaccinees, it was due to the increase of CD4^+^ T cells (Figure 4A), whereas in OBS subjects it was due to the decrease of CD8^+^ T cells (Figure 4C), as reported earlier to occur during HAART [54-56].

**Figure 7:**
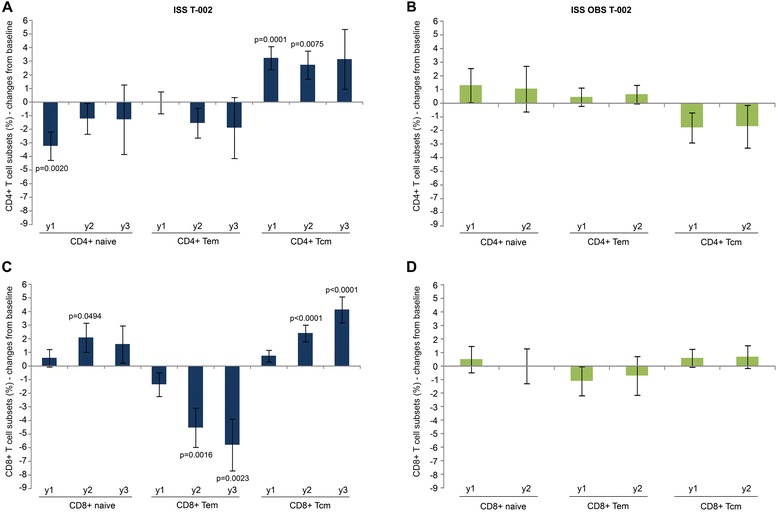
**Naïve, central and effector memory CD4** ^**+**^ **and CD8** ^**+**^ **T cells.** Changes from baseline of naïve, Tem and Tcm **(A, B)** CD4^+^ and **(C, D)** CD8^+^ T cell percentage at years 1, 2 and 3 for vaccinees (left panels) (n = 94 at year 1 ; n = 75 at year 2 ; n = 32 at year 3) and OBS subjects (right panels) (n = 32 at year 1 ; n = 20 at year 2). Data are presented as mean values with standard error. A longitudinal analysis for repeated measurements was applied. P-values assess the values at year 1, 2 or 3 after immunization versus baseline values.

In vaccinees, changes in CD4^+^ and CD8^+^ T cells were accompanied by an early, durable and statistically significant restoration of functional subsets, while few or no changes were observed in OBS subjects (Figure 7). In particular, early (year 1) and durable (up to year 3) increases of CD4^+^ central memory T cells (Tcm) occurred in vaccinees. This was associated with decreases of naïve and effector memory T cells (Tem), which, however, in most cases did not reach statistical significance (Figure 7A). No significant changes from baseline of CD4^+^ T cell subsets were seen in OBS subjects (Figure 7B) as reported in other studies [52,53].

In vaccinees changes in the CD8^+^ T cell compartment followed those seen in the CD4^+^ T cell subsets. In particular, statistical significant increases of CD8^+^ naïve and Tcm cells with a concomitant decrease of the Tem subset were observed at year 2 and/or year 3 (Figure 7C), whereas, as reported earlier in treated subjects [46,53], no significant changes were noticed in OBS subjects (Figure 7D).

**Figure 8:**
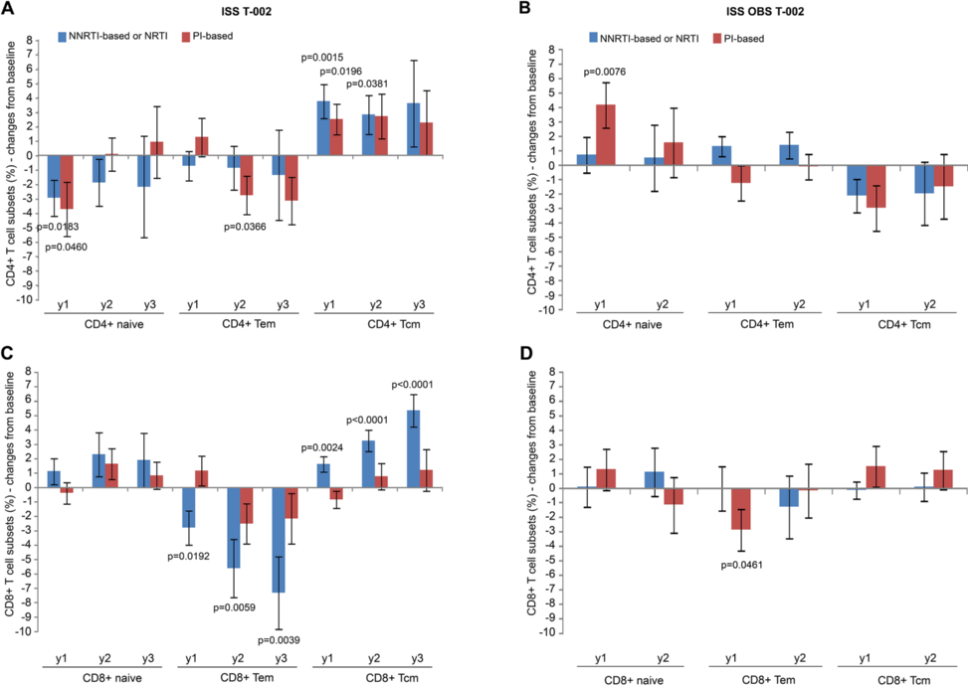
**Naïve, central and effector memory CD4** ^**+**^ **and CD8** ^**+**^ **T cells stratified by antiretroviral regimens.** Changes from baseline of naïve, Tem and Tcm **(A, B)** CD4^+^ and **(C, D)** CD8^+^ T-cell percentage at years 1, 2 and 3 according to antiretroviral regimens in vaccinees (left panels) (NNRTI- or NRTI-based n = 59 at year 1 ; n = 49 at year 2 ; n = 21 at year 3; PI-based n = 35 at year 1 ; n = 26 at year 2 ; n = 11 at year 3) and OBS subjects (right panels) (NNRTI- or NRTI-based n = 19 at year 1 ; n = 9 at year 2; PI-based n = 13 at year 1 ; n = 11 at year 2). Data are presented as mean values with standard error. A longitudinal analysis for repeated measurements was applied. P-values assess the values at year 1, 2 or 3 after immunization versus baseline values, both overall and stratified by drug regimens (NNRTI/NRTI- or PI-based).

Similar results were observed in vaccinees with both drug regimens (NNRTI/NRTI- or PI-based) for the CD4^+^ T cell subsets (Figure 8A), whereas for the CD8^+^ T cell subsets NNRTI were associated with greater and significant decreases of CD8^+^ Tem and increases of CD8^+^ Tcm as compared to PI-based regimens (Figure 8C). In OBS subjects PI-based regimens had more pronounced effects on CD4^+^ and CD8^+^ T cell subsets, particularly for the increase of naïve CD4^+^ T cells (Figure 8B), and for the reduction of CD8^+^ Tem at year 1, whereas NNRTI-based regimens had no effects (Figure 8D). Thus, Tat immunization restores CD4^+^ and CD8^+^ T cell subsets during HAART; however, differences are seen according to the drug regimens, particularly for the CD8^+^ T cell subsets which are better restored with NNRTI-based regimens.

### HIV proviral DNA

**Figure 9:**
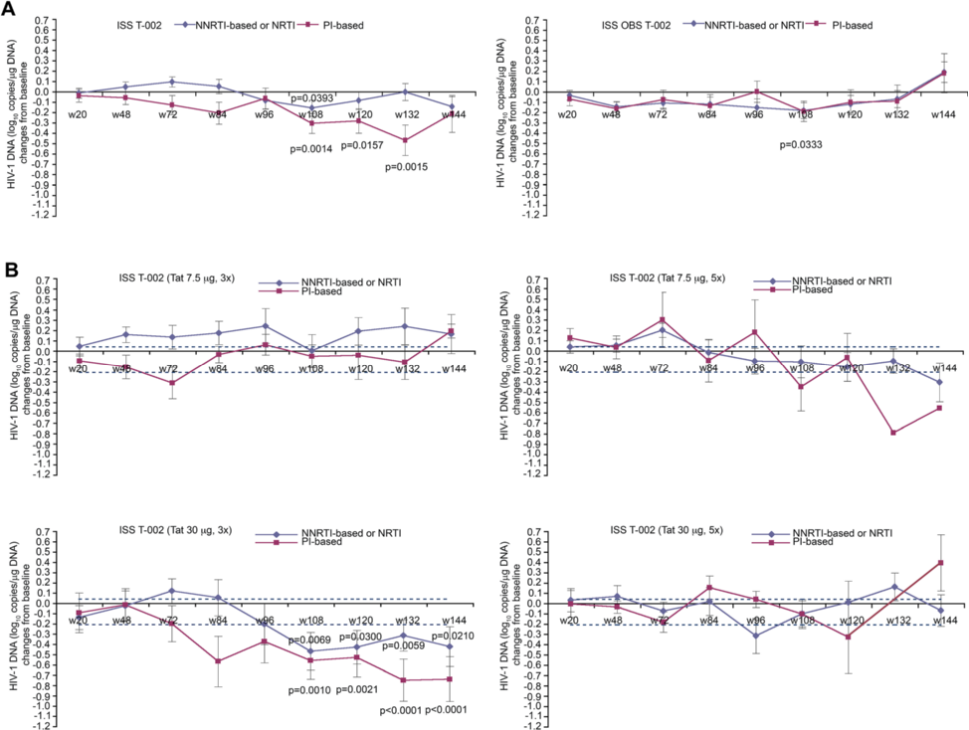
**Blood HIV-1 DNA load (expressed as log**_**10**_ **copies/μg DNA) stratified by antiretroviral regimens. (A)** Changes of HIV-1 DNA (expressed as log_10_ copies/μg DNA) versus baseline according to antiretroviral regimens in vaccinees (NNRTI- or NRTI-based n = 102; PI-based n = 45) or OBS subjects (NNRTI- or NRTI-based n = 36; PI-based n = 26). **(B)** Changes of HIV-1 DNA versus baseline in vaccinees of each treatment group stratified according to ARV regimens (7.5 μg, 3x n = 38; 7.5 μg, 5x n = 37; 30 μg, 3x n = 37; 30 μg, 5x n = 35). The dotted lines represent the 99% confidence interval of HIV-1 DNA mean change from baseline (−0.21, 0.04 log_10_ copies/μg) in OBS subjects (all time points). In all panels data are presented as mean values with standard error. Median time of follow-up was 96 weeks for vaccinees and 120 weeks for OBS subjects. A longitudinal analysis for repeated measurements was applied. P-values assess the values at each week after immunization versus baseline values stratified by drug regimens (NNRTI/NRTI- or PI-based).

HIV-1 DNA was evaluated longitudinally up to 3 years (144 weeks) in vaccinees and OBS subjects prior to or after stratification by drug regimens. As shown in Figure 9A, although they had equal baseline mean values (1.2 log_10_ copies/μg DNA), a reduction of HIV-1 DNA was seen in vaccinees as compared to OBS subjects, particularly under PI-based regimens. When treatment groups were analyzed separately (Figure 9B), Tat 30 μg, 3x showed the highest, most persistent and statistically significant HIV-1 DNA reduction with both drug regimens, although a greater decay was observed again with PI-based regimens. In particular, the percentage of reduction in all vaccinees under PI-based regimens (Figure 9A), when statistically significant, ranged from 51% (week 108) to 66% (week 132), and in the Tat 30 μg, 3x group from 52% (week 108) to 65% (week 144) (Figure 9B).

**Figure 10:**
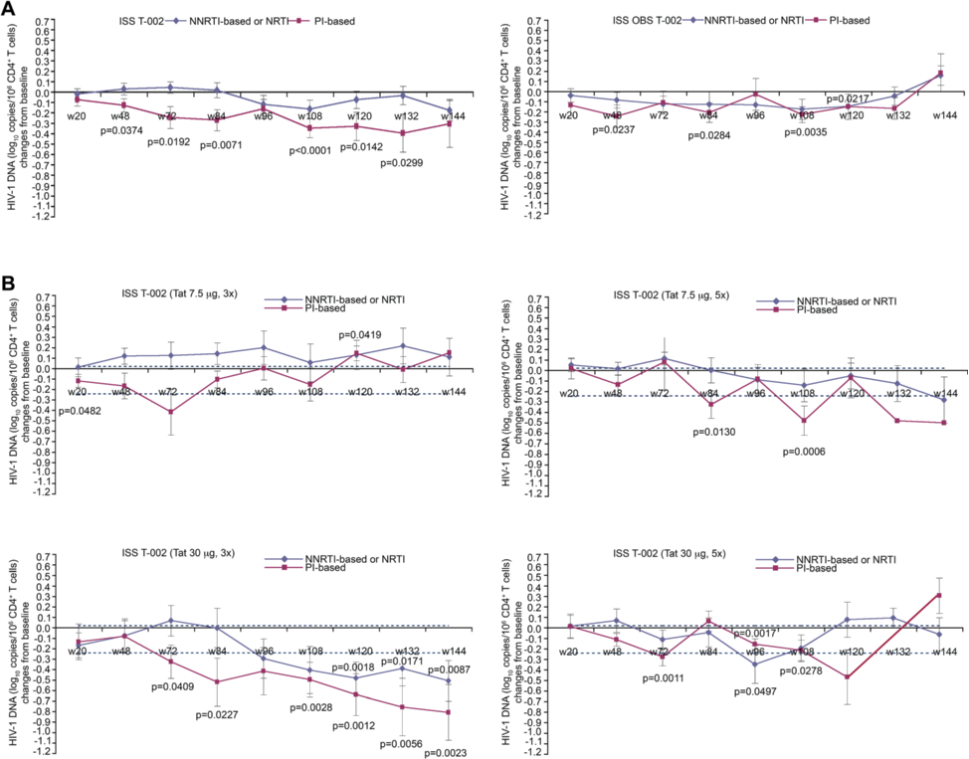
**Blood HIV-1 DNA load (expressed as log**_**10**_ **copies/10** ^**6**^ **CD4** ^**+**^ **T cells) stratified by antiretroviral regimens. (A)** Changes of HIV-1 DNA (expressed as log_10_ copies/10^6^ CD4^+^ T cells) in vaccinees (NNRTI- or NRTI-based n = 97; PI-based n = 45) or OBS subjects (NNRTI- or NRTI-based n = 30; PI-based n = 24). **(B)** Changes of HIV-1 DNA in vaccinees of each treatment group stratified according to ARV regimens (7.5 μg, 3x n = 36; 7.5 μg, 5x n = 36; 30 μg, 3x n = 36; 30 μg, 5x n = 34). The dotted lines represent the 99% confidence interval of HIV-1 DNA mean change from baseline (−0.24, 0.02 log_10_ copies/10^6^ CD4^+^ T cells) in OBS subjects (all time points). In all panels data are presented as mean values with standard error. Median time of follow-up was 96 weeks for vaccinees and 120 weeks for OBS subjects. A longitudinal analysis for repeated measurements was applied. P-values assess the values at each week after immunization versus baseline values stratified by drug regimens (NNRTI/NRTI- or PI-based).

Since CD4^+^ T cells harbor most of the provirus detectable in blood [46], HIV-1 DNA was then calculated according to CD4^+^ T cell counts. As shown in Figure 10A, the percentage of proviral reduction in all vaccinees under PI-based regimens ranged from 26% (week 48) to 60% (week 132), and in the Tat 30 μg, 3x under PI-based regimens ranged from 53% (week 72) to 85%, (week 144), followed by Tat 7.5 μg, 5x (range from 53% to 67%, at week 84 and 108, respectively) and Tat 30 μg, 5x (range from 31% to 47%, at week 96 and 72, respectively) (Figure 10B). Conversely, lower and transient reductions were seen in the 7.5 μg, 3x group and in OBS subjects, and were mostly observed under PI-based regimens. The changes of proviral load in OBS recapitulate what previously reported under successful therapy [59,60].

**Figure 11:**
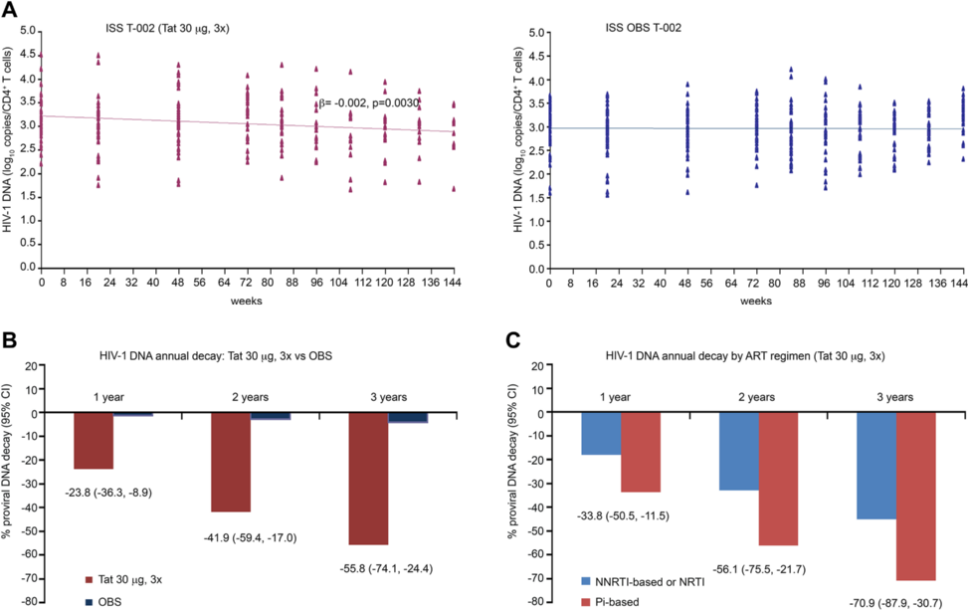
**HIV-1 DNA decay in individuals immunized with Tat at 30 μg, 3x or OBS subjects. (A)** Longitudinal regression analysis of HIV-1 DNA (log_10_ copies/10^6^ CD4^+^ T cells) using a random-effect regression model up to 144 weeks since the first immunization in vaccinees (Tat 30 μg, 3x, n = 37) or OBS subjects (n = 62). All longitudinal data were included in the analysis with a median of 108 weeks of follow-up in vaccinees and 120 weeks in OBS subjects, respectively. **(B)** Estimates of HIV-1 DNA decay based on the regression model after 1, 2 and 3 years since the first immunization in vaccinees (Tat 30 μg, 3x in red) and in OBS subjects (in blue). **(C)** Estimates of HIV-1 DNA annual decay in vaccinees immunized with Tat 30 μg, 3x stratified according to NNRTI- or NRTI-based (in blue, n = 25) or PI-based (in red, n = 12) regimens. Results in panels B and C are expressed as the percentage of HIV-1 DNA decay with 95% confidence interval (CI).

The longitudinal regression analysis confirmed the HIV-1 DNA decay in the Tat 30 μg, 3x group, but not in OBS subjects, with an estimate of 56% reduction after 3 years from vaccination (Figure 11A, B). The decay was more pronounced under PI-based regimens for which the estimate reached over 70% reduction after 3 years, with a half-life of 88 weeks (Figure 11C).

HIV-1 DNA decay was then analyzed versus anti-Tat humoral responses adjusted for treatment groups and antiretroviral therapy by a multivariate regression model (Table 8). The greatest and most significant HIV-1 DNA reduction occurred in the presence of both anti-Tat IgM and IgG Abs at year 2 and, particularly, at year 3 from vaccination, whereas in the presence of a single anti-Tat Ab isotype, it was observed only in year 3. Anti-Tat Ab negative vaccinees had a reduction of borderline significance at year 3, possibly explained by the fact that most of these subjects (76%) had Tat-specific cellular responses. In fact, anti-Tat Ab negative but anti-Tat cellular positive subjects showed a decrease of HIV-1 DNA both in year 2 and 3 from vaccination (mean: −0.12 and −0.16 log_10_ copies/10^6^ CD4^+^ T cells, representing 24% and 31% reduction, respectively), whereas a slight increase was detected in subjects with no humoral or cellular responses to Tat (0.1 log_10_ copies/10^6^ CD4^+^ T cells) (Table 9). HIV DNA changes similar to those observed in anti-Tat Ab negative vaccinees were also seen in OBS subjects according to the presence or absence of Tat-specific cellular responses (Table 9). Further, the 30 μg, 3x vaccine regimen induced the highest HIV-1 DNA decay, however, also the 7.5 μg, 5x showed statistically significant HIV-1 DNA decreases at year 3, whereas the 30 μg, 5x showed a trend only at year 2, whereas no significant changes were observed in 7.5 μg, 3x group (Table 8). Finally, Tat vaccination in association with PI-based regimens resulted again the most effective at reducing HIV-1 DNA (Table 8).

**Table 8:** **HIV-1 DNA vs anti-Tat humoral immune responses, adjusted for treatment groups and antiretroviral drug regimens**

**HIV-1 DNA (log**_**10**_ **copies/10** ^**6**^ **CD4** ^**+**^ **)**	**Year** ^**(a)**^	**Estimate** ^**(b)**^	**95% CI** ^**(c)**^	**p value**
*Anti-Tat humoral response*				
IgM^+^ and IgG^+^	2^nd^	−0.17	−0.28; −0.05	0.0036
	3^rd^	−0.26	−0.41; −0.10	0.0012
IgM^+^, IgG^+^ or IgA^+^	2^nd^	0.02	−0.08; 0.12	0.3837
	3^rd^	−0.17	−0.32; −0.01	0.0356
Ab Negative	2^nd^	−0.13	−0.30; 0.04	0.1345
	3^rd^	−0.23	−0.46; −0.00	0.0546
*Treatment groups*				
Tat 7.5 μg, 3x	2^nd^	−0.01	−0.14; 0.12	0.8997
	3^rd^	0.03	−0.18; 0.25	0.7572
Tat 7.5 μg, 5x	2^nd^	−0.10	−0.17; 0.03	0.1794
	3^rd^	−0.25	−0.43; −0.10	0.0057
Tat 30 μg, 3x	2^nd^	−0.16	−0.35; 0.03	0.0975
	3^rd^	−0.53	−0.73; −0.33	<0.0001
Tat 30 μg, 5x	2^nd^	−0.13	−0.29; 0.02	0.0887
	3^rd^	−0.12	−0.31; 0.07	0.2156
Tat 30 μg, 3x vs Tat 7.5 μg, 3x	3^rd^	−0.56	−1.02; −0.10	0.0050
*Antiretroviral regimens*				
NNRTI-based or NRTI		−0.05	−0.16; 0.05	0.2971
PI-based		−0.25	−0.36; −0.15	<0.0001
NNRTI-based or NRTI vs PI-based		0.20	0.06; 0.34	0.0047

^(a)^Year since first immunization: 48, 72, 84, 96 weeks (2^nd^ year); 108, 120, 132, 144 weeks (3^rd^ year); ^(b)^Least square means; ^(c)^confidence interval.

Multivariate analysis of variance for repeated measures of longitudinal samples from 136 individuals.

**Table 9:** **Anti-Tat cellular response according to anti-Tat Abs and HIV-1 DNA vs anti-Tat immune responses**

	**n/N** ^(b)^	**Percentage**	
*Anti-Tat cellular response in vaccinees* ^*(a)*^			
IgM^+^ and IgG^+^	60/67	89.5	
IgM^+^, IgG^+^ or IgA^+^	34/40	85.0	
Ab Negative	22/29	75.9	
**HIV-1 DNA (log**_**10**_ **copies/10** ^**6**^ **CD4** ^**+**^ **T cells)**	**Year** ^(c)^	**Mean ± s.e.** ^(d)^	**n**
Vaccinees			
*Anti-Tat Ab negative and cellular positive*			
	2^nd^	−0.08 ± 0.09	21
	3^rd^	−0.21 ± 0.11	11
	2^nd^ and 3^rd^	−0.08 ± 0.08	22
*Anti-Tat Ab negative and cellular negative*			
	2^nd^	0.09 ± 0.14	7
	3^rd^	0.10 ± 0.57	3
	2^nd^ and 3^rd^	0.06 ± 0.17	7
OBS subjects^*(e)*^			
*Anti-Tat Ab negative and cellular positive*			
	2^nd^	−0.12 ± 0.07	37
	3^rd^	−0.16 ± 0.07^(*)^	28
	2^nd^ and 3^rd^	−0.13 ± 0.07	37
*Anti-Tat Ab negative and cellular negative*			
	2^nd^	−0.07 ± 0.07	8
	3^rd^	0.09 ± 0.12	8
	2^nd^ and 3^rd^	0.00 ± 0.05	8

^(a)^Cumulative anti-Tat cellular responses up to 48 weeks in vaccinees. Anti-Tat cellular responses included IFN-γ, IL**-**4, and IL**-**2 production and CD4^+^ or CD8^**+**^ T cell proliferative responses.

^(b)^n = number of subjects positive for anti-Tat cellular responses; N = total number of evaluated subjects. ^(c)^Year since first immunization: 48, 72, 84, 96 weeks (2^nd^ year); 108, 120, 132, 144 weeks (3^rd^ year). ^(d)^s.e. = standard error; ^(*)^ p = 0.0426, Student’s *t-*test for paired data. ^(e)^The frequency of anti-Tat cellular responses in OBS subjects was 45/61 (74%).

### Neutralization of Tat-mediated Env entry in dendritic cells

**Figure 12:**
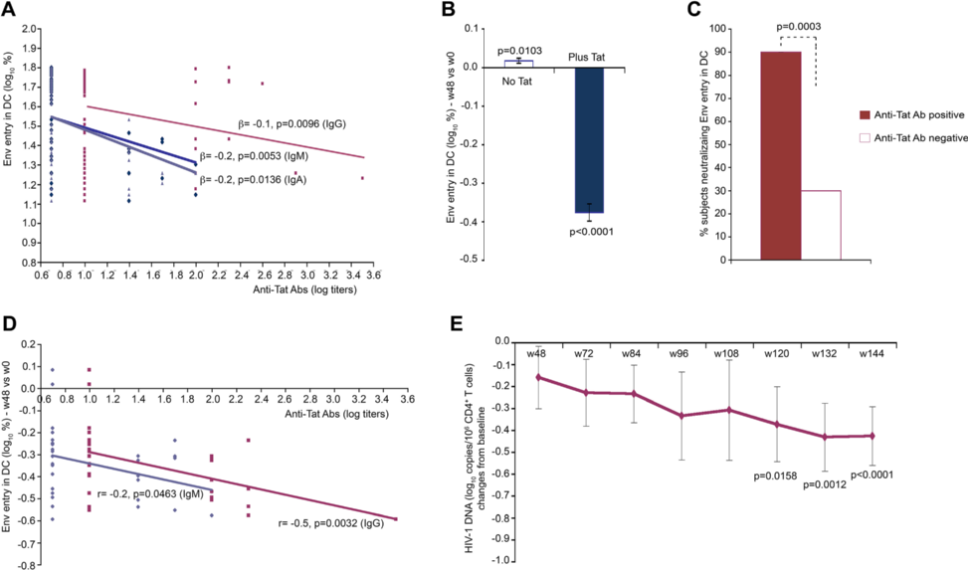
**Neutralization of Tat-mediated Env entry in DC and HIV-1 DNA in vaccinees. (A)** Relationship between Tat-mediated entry of trimeric Env in DC and anti-Tat IgM (blue), IgG (red) and IgA (violet) Ab binding titers in subjects immunized with Tat 30 μg, 3x (generalized estimating equations with adjustment for repeated measures in the same patient; longitudinal samples from 31 individuals). **(B)** Percentage of Env entry in DC in the presence or absence of Tat in subjects immunized with Tat 30 μg, 3x at 48 weeks since the first immunization (n = 32) (Student’s *t*-test). Data are expressed as mean changes from baseline with standard error. **(C)** Inhibition of Tat-mediated Env entry in DC by anti-Tat Ab positive (n = 20) and anti-Tat Ab negative (n = 12) sera at week 48 from subjects immunized with Tat 30 μg, 3x. Of the 32 subjects evaluated, 30 were positive for anti-Tat Abs before week 48. Data are expressed as the percentage of subjects showing neutralization of Tat-mediated Env entry at 48 weeks as compared to baseline. (Fisher's Exact test) **(D)** Pearson correlation between anti-Tat IgM and IgG Ab titers at 48 weeks and Tat-mediated Env entry in DC at 48 weeks versus baseline in subjects immunized with Tat 30 μg, 3x (n = 32). **(E)** Reduction from baseline of HIV-1 DNA (log_10_ copies/10^6^ CD4^+^ T cells) over time in sera from vaccinees (Tat 30 μg, 3x) positive for neutralization (≥50%) of Env entry in DC at week 48 since vaccination (n = 20)_._ Data in panels are presented as mean values with standard error. Changes from baseline of HIV-1 DNA in subjects with anti-Tat Abs and neutralizing activity were evaluated with a longitudinal analysis for repeated measurements. P-values assess the values at each week after immunization versus values at baseline.

Since the Tat 30 μg, 3x regimen was the most effective at reducing HIV DNA, neutralization of the entry of oligomeric Env into DC in the presence or absence of Tat [32] was assessed in this treatment group by analyzing sera at week 0 (baseline) and at week 12, 20 and 48 after immunization. This assay overcomes the problem posed by HAART interference with neutralization of HIV-1 infection [61]. Sera blocking Tat-mediated Env entry in DC by ≥50% versus baseline were indicated as neutralizing. A significant inverse relationship was observed between Tat-mediated Env entry in DC and anti-Tat Ab binding titers of all isotypes (Figure 12A), particularly at week 48 (Figure 12B). At this time, neutralization was observed in 90% of the subjects with anti-Tat Abs (Figure 12C) and correlated with both anti-Tat IgM and IgG titers (Figure 12D). Of note, the presence of anti-Tat Abs and neutralizing activity at week 48 predicted a significant reduction of HIV-1 DNA, which was consistently detected at year 2 and particularly at year 3 (Figure 12E).

### Induction of effector memory CD38^+^ HLA-DR^+^/CD8^+^ T cells and restoration of NK cells

The data described above indicated that proviral reduction is observed late after immunization, is greatest in the 30 μg, 3x group, and under PI based regimens, is associated with the presence of IgM and IgG anti-Tat Abs, and is predicted by the neutralization of virus entry at year 1.

**Figure 13:**
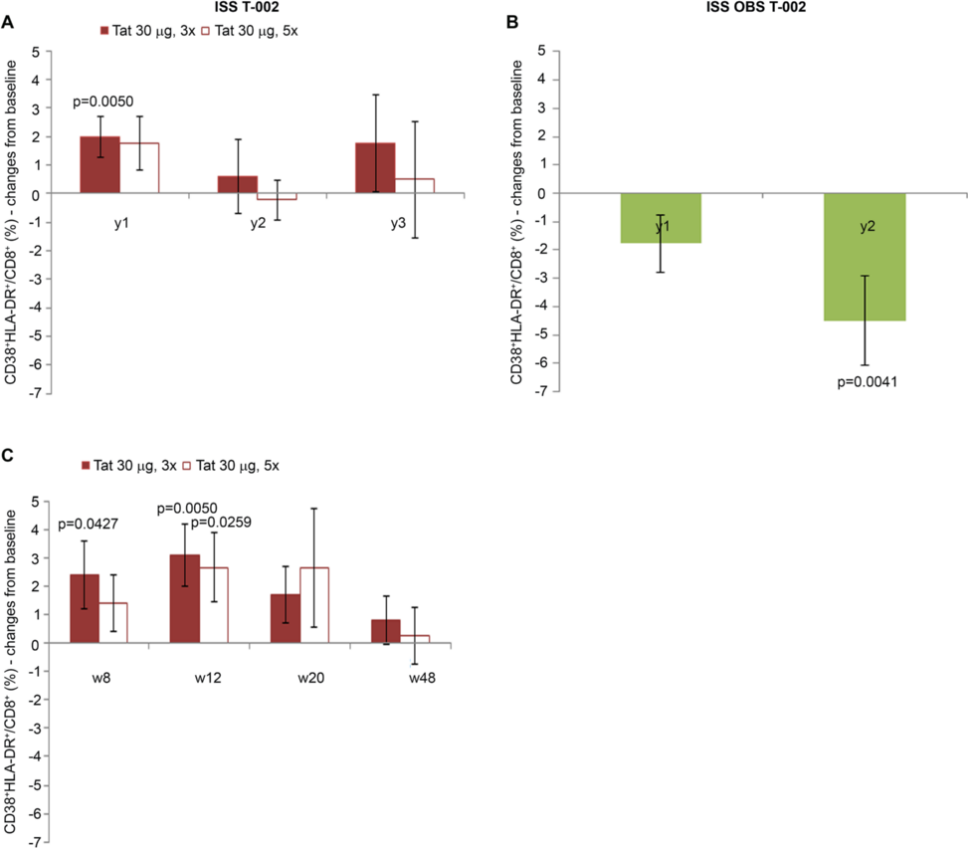
**CD38** ^**+**^ **HLA-DR** ^**+**^ **/CD8** ^**+**^ **T cells in individuals immunized with Tat at the 30 μg doses or in OBS subjects.** Changes from baseline of CD38^+^HLA-DR^+^/CD8^+^ at years 1, 2 and 3 in **(A)** vaccinees of the Tat 30 μg, 3x group (year 1: n = 28; year 2: n = 20; year 3: n = 10) and the Tat 30 μg, 5x group (year 1: n = 28; year: 2 n = 19; year 3: n = 9); **(B)** OBS subjects (year 1: n = 38; year 2: n = 25). **(C)** Changes from baseline of CD38^+^HLA-DR^+^/CD8^+^ at week 8, 12, 20 and 48 in vaccinees of the Tat 30 μg, 3x group (n = 28); and the Tat 30 μg, 5x group (n = 28). Data are presented as mean values with standard error. A longitudinal analysis for repeated measurements was applied. P-values assess the values at year 1, 2, or 3 or week 8, 12, 20 or 48 after immunization versus baseline values.

**Figure 14:**
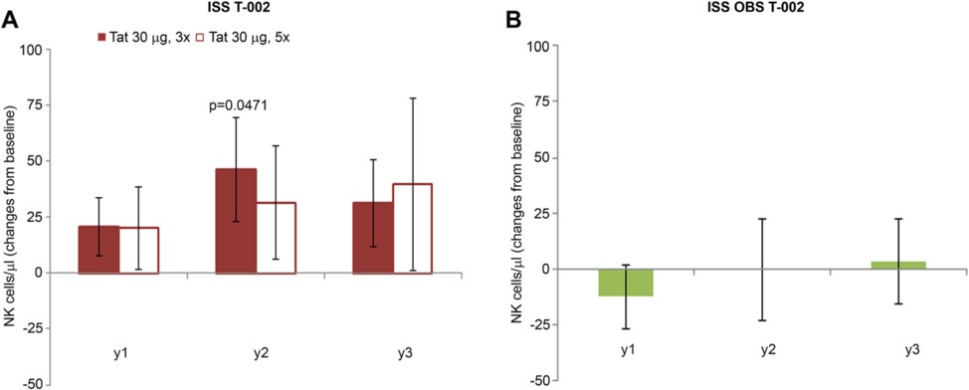
**NK cells in immunized with Tat at the 30 μg doses or in OBS subjects.** Changes from baseline of NK cells/μl at years 1, 2 and 3 in **(A)** vaccinees of the Tat 30 μg, 3x (year 1 n = 37; year 2 n = 27; year 3: n = 18); and the Tat 30 μg, 5x group (year 1: n = 37; year 2: n = 26; year 3: n = 16); and **(B)** OBS subjects (year 1: n = 79; year 2: n = 42; year 3: n = 30). Data are presented as mean values with standard error. A longitudinal analysis for repeated measurements was applied. P-values assess the values at year 1, 2 or 3 after immunization versus baseline values.

In particular, the presence of IgG and IgM anti-Tat Abs was more frequent in both the 30 μg dose regimens (60% in 30 μg, 3x group and 62% in 30 μg, 5x group) (Figure 2D), although proviral reduction was greater when Tat was given 3 times as compared to 5 times. Therefore, all immunological parameters for which data were available were compared between the two treatment groups. As shown in Figure 13 and Figure 14, the only relevant differences were found for CD38^+^ HLA-DR^+^/CD8^+^ T cell percentage and NK cell number. In particular, in the 30 μg, 3x group, CD38^+^ HLA-DR^+^/CD8^+^ T cells were significantly increased at year 1, whereas only a trend was seen in the 30 μg, 5x group, and thereafter these cells declined in both groups, particularly in the 30 μg, 5x regimen (Figure 13A). Flow cytometry data analysis indicated that the increase of CD38^+^HLA-DR^+^/CD8^+^ T cells was due to the upregulation of HLA-DR expression, a marker associated with proliferative capacity and found increased in elite controllers [62]. Stratification of year 1 data by weeks indicated that the increase of this cell phenotype occurred and was significant in both groups after 3 immunizations (week 12) (Figure 13C), whereas further immunizations in the 30 μg, 5x group did not increase or maintain these cells, which, in contrast, were reduced down to undetectable levels at 48 weeks (Figure 13C). Significant reductions were observed in OBS subjects upon time (Figure 13B).

Concerning NK cells, the 30 μg, 3x group showed significant increases at year 2, whereas not significant increases were seen in the 30 μg, 5x group (Figure 14A), and few or no changes were observed in OBS subjects (Figure 14B).

Thus, the 30 μg, 3x regimen had the highest frequency of effector cells (CD38^+^HLA-DR^+^/CD8^+^ and NK cells), endowed with killing capacity against virus-infected cells [62]. Of note the CD38^+^ HLA-DR^+^ CD8^+^ T cells are effector memory CD8^+^ T cells found to be increased in elite controllers [62].

## Discussion

Several strategies have been undertaken to intensify HAART efficacy in order to attack the virus reservoirs, reduce immune activation and to restore functional T, B and NK cells [9]. However, they have been unsuccessful or have had only a marginal effect [9,63].

The results of the ISS T-002 reported here not only confirm previous findings of safety and immunogenicity of the Tat vaccine [22,26-28], but also show that immunization with Tat in HAART-treated, virologically-suppressed subjects increases T, B and NK cell numbers and promotes restoration of CD4^+^ and CD8^+^ functional T cell subsets toward homeostasis. This is not observed in the external reference group, which recapitulates what is reported in the literature after years of HAART [1].

Immune restoration started early after vaccination, increased upon time and was durable (up to 3 years). In particular, a mean increase of CD4^+^ T cells up to values of about 100 cells/μl was observed in vaccinees at year 3 (Figure 4A), which appears highly meaningful in subjects with a mean of 6 years of therapy. In fact, a successful HAART regimen is expected to induce increments of CD4^+^ T cells of about 50 cells/month in the first 3 months since therapy initiation and then of approximately 50 cells/year in the next 3 years or so. Then CD4^+^ T cells plateau, remaining generally stable thereafter [51-53] as observed in OBS subjects (Figure 4B). Further, in vaccinees CD4^+^ T cell number increased progressively independently from the nadir (Figure 6A). This is of clinical relevance since it is known that subjects with a nadir ≤250 cells/μl do not respond as well to therapy [52,53].

Taken together these immunological changes appear consistent with a restoration of immune functions, well beyond what is achieved upon cART, in which, for example, CD4^+^ T cell counts are incompletely restored, and CD4^+^ and CD8^+^ central memory subsets remain underrepresented, due to the residual immune activation which fuels the transition from naive to effector cells and hampers the transition to the central memory phenotype generally occurring in the contraction phase of an immune response (reviewed in [64,65]). In this regard the increase of central memory CD4^+^ T cells and the reduction of effector memory CD8^+^ T cells in association with the expansion of central memory CD8^+^ T cells appear of particular relevance (Figure 7).

Concerning other therapeutic approaches including HIV vaccines tested so far in humans (reviewed in [66-68]), it should be underscored that in most vaccine trials therapy was interrupted to assess the potency of the immunological control of infection provided by vaccination, and these studies were not aimed at evaluating the immunological recovery with the exclusion of the CD4^+^ T cell counts, which, however, do not appear to go beyond the restoration provided by cART alone (reviewed in [67,68]).

In this contest of T cell immune restoration, an increase of B (Figure 4 E, F) and NK cells (Figure 4 G,H) was detected in vaccinees after a mean of 6 years of therapy. This was not observed in OBS subjects and it is not achieved with other therapeutic approaches [57,58]. Of note, B cell numbers at baseline were significantly lower in vaccinees as compared to OBS subjects, although well within the normal range in both groups. However, opposite kinetics were observed upon time in the two groups, with vaccinees experiencing a moderate (about 30–40 cell/μL) but durable (3 years) and statistical significant (with respect to the baseline levels) expansion of the absolute B cell counts, whereas an opposite, non-significant, trend was observed in OBS subjects over the two years of follow up. This difference with respect to the baseline levels recorded for the T-002 trial would argue against a simple leveling off of total B cell counts, eventually leading to comparable absolute counts in the two studies at the end of follow-up.

Although Tat immunization promoted similar effects with both drug regimens, NNRTI-based regimens were the most effective at restoring CD8^+^ T cell subsets.

Overall, all treatment groups experienced, although to a different extent, immune restoration with the 7.5 μg, 3x performing the least.

Immune restoration was followed by a significant and progressive HIV-1 DNA decay in blood, particularly with Tat 30 μg, 3x under PI-based regimens, which was associated with the presence of both anti-Tat IgM and IgG Abs and Env neutralization, that predicted HIV-1 DNA decay. In addition, only the 30 μg, 3x group showed significant increases of effector memory CD38^+^ HLA-DR^+^/CD8^+^ T cells and NK cells, which are known to exert killing activities against virus infected cells (reviewed in [69,70]). In particular, significant increases of CD38^+^ HLA-DR^+^/CD8^+^ T cells were observed in both 30 μg regimens after 3 immunizations and were associated with peak IgM and IgG anti-Tat Ab titers. However, additional immunizations in the 30 μg, 5x group did not increase or maintain but, in contrast, further reduced this cell phenotype, supporting the concept that too many vaccine inocula may initiate suppressive regulatory mechanisms likely by overstimulation of the immune system [71,72].

The risk to hamper a good antiviral response by immune overstimulation is particularly true for Tat, which is an active molecule, capable of targeting and entering efficiently key antigen-presenting cells (i.e. DC), inducing cell activation and maturation [24,42]. In addition, since the vaccine is administered intradermally and the derma is very rich in DC, higher doses and/or repeated immunizations may easily induce an overstimulation, modifying vaccine-induced immune responses [73,74]*.* Thus, the 30 μg, 3x regimen appears to be the best one to achieve both immune restoration and antiviral responses.

All the analyses shown herein were performed in the immunogenicity population as defined in the clinical protocol, however, data (anti-Tat Abs, CD4^+^ T cells and HIV proviral DNA) did not change by utilizing an “intention-to-treat” analysis.

Taken together, these data indicate that Tat immunization induces key antiviral activities likely due to multiple mechanisms. In particular, anti-Tat Abs can block the Tat/Env complex formation and virus entry into DC and other integrin-expressing cells of the reticular-endothelial system [32]. These cells represent the first HIV-1 targets at the portals of entry in primary infection [75,76], and become HAART-resistant virus reservoirs in chronic infection [1-3,77-79]. Since HIV-1 sera regain neutralizing activity in the presence of anti-Tat Abs, it is likely that these Abs block the ongoing cell-to-cell virus transmission [6], thus impeding replenishment of the virus reservoir. This then progressively reduces owing to the turnover of infected CD4^+^ T cells [48,80], as suggested by the late proviral decay and its kinetics. In this regard, the restoration of functional T cell subsets and NK cells seen early after Tat vaccination may render more effective immune recognition and clearance of infected cells [48], effectively contributing to proviral DNA decay. In particular, the induction of both CD38^+^ HLA-DR^+^/CD8^+^ T cells, which have been demonstrated to exert effective cytotoxic antiviral activity in elite controllers [62], and NK cells suggest that these effector cells may contribute to proviral reduction. However, at present a formal proof of the killing activity of these cells in vaccinees is not available.

A limitation of this study is that it was not a double-blinded, placebo-controlled trial. However, this decision was made to have the possibility of exploring in depth the effect of Tat vaccination on biomarkers of disease, with the aim of including additional analyses if deemed necessary to better understand mechanism(s) of action and to define the proper biomarkers for future trials. To this goal an external reference group (OBS) was used following the recommendations of regulatory guidelines [50]. This group was not used as a control of the pre-specified endpoints of the protocol (immunogenicity/safety) but served only to provide a side-by-side view of the immunological and virological disease biomarkers in anti-Tat Ab negative subjects after years of effective HAART without any direct comparison with the trial subjects, since they belong to two separate studies. Indeed, in the OBS subjects the results for all the immunological and virological parameters analyzed are consistent with those reported in the literature in successfully treated patients after years of HAART [1].

The open label design of the trial permitted to verify the rapid achievement of the study primary and secondary endpoints and to appreciate the immune reconstitution induced by vaccination [22]. This allowed the implementation of the study protocol with proper amendments, such as inclusion of more immune compromised patients who, in fact, showed the most pronounced immunological benefit. Of note, this approach also allowed the design and the definition of the proper laboratory testing of a placebo-controlled double-blinded confirmatory phase II study of the Tat vaccine at 30 μg, 3x (ISS T-003, Clinicaltrials.gov NCT01513135), which has been successfully completed in 200 individuals on effective HAART in South Africa.

## Conclusions

The results indicate that the Tat vaccine candidate is safe and immunogenic. In addition, they suggest that Tat-induced immune responses are necessary to restore immune homeostasis and effective antiviral activities. However, results from the trial just completed in South Africa and further efficacy studies will be needed to confirm these findings as well as to address issues of HIV subtype variability, and other problems of virus variability, to prove vaccine efficacy in other HIV clade infections. Therefore, at this time, no definitive conclusions can be drawn regarding the ultimate viability of this vaccine approach, which has been considered controversial by other groups. Nevertheless, the findings in this study may help establishing key parameters for HAART intensification and a functional cure.

## Methods

### Study design and participants

The phase II therapeutic trial with the biologically active HIV-1 Tat protein (ISS T-002) was a multicenter, randomized, open label trial (ISS T-002, Clinicaltrials.gov NCT00751595) designed to evaluate immunogenicity and safety, and to explore immunological and virological biomarkers of disease (second-line testing). Tat was administered at 7.5 μg or 30 μg, given 3 (3x) or 5 (5x) times to 168 HIV-1 infected HAART-treated adults, anti-Tat Ab negative at baseline with undetectable levels of plasma viremia (<50 copies/mL) in the last 6 months prior to enrollment, and CD4^+^ T-cell number ≥200 cells/μL. The trial was closed at week 48 after the first immunization and a subgroup of patients was followed up to 3 years (n = 76 up to 96 weeks and n = 45 up to 144 weeks). A group of 79 anti-Tat Ab negative HAART-treated subjects from a parallel observational (OBS) study (ISS OBS T-002, Clinicaltrials.gov NCT01024556) represented the external reference group for disease biomarkers according to ICH guidelines [50]. In particular, the potential bias of an external nonrandomized control group was limited according to recommendations of the regulatory guidelines by enrolling subjects in the same clinical centers, with the same inclusion criteria of the trial, by using the same procedures, and by simultaneous evaluations by the same core laboratory. This group recapitulates what is usually observed in individuals after years of effective HAART [1-7,46,51-58].

### Study outcomes

The *primary outcome* of the study (immunogenicity) was measured by the induction, magnitude and persistence of the humoral immune responses to Tat. Cellular immune responses to Tat were a co-primary endpoint. The anti-Tat humoral immune response was evaluated by determination and titration of IgM, IgG and IgA anti-Tat Abs in sera. The anti-Tat cellular immune response was measured in blood by the assessment of CD4^+^ and CD8^+^ lymphoproliferative responses, and production of IFN-γ, IL-4 and IL-2 in response to Tat, as detailed below.

The *secondary outcome* of the study (safety) was assessed by the collection of all adverse events occurred during the trial, including vital signs monitoring, and any clinically significant change in hematological, biochemical and coagulation parameters. The study was completed at 48 weeks according to a protocol amendment approved by Ethic Committees (see below), vaccinees were followed up until the last enrolled subjects reached the 48 weeks. Therefore, the safety results are shown up to study closure (48 weeks), but also described for the entire follow-up (144 weeks). All randomized subjects who received at least one administration of the vaccine (168 subjects) were included in the safety data analysis. All AE were classified according to the preferred term and the system organ class (SOC) of the Medical Dictionary for Regulatory Activities (MedDRA) as well as on the basis of the drug relationship and grade of severity. Safety data were periodically evaluated during the study by the DSMB and Data Safety Update Reports were submitted to the Competent Authorities, according to the current regulatory requirements.

### Sample size and randomization

As the primary objective of the study protocol was the immunogenicity of the Tat vaccine, sample size was estimated separately in each vaccination arm. To observe a proportion of subjects with a humoral immune response to vaccination (primary endpoint) within at least 75%-80%, considering a margin of error of 12%-13% and a confidence level of 95%, at least 40 evaluable subjects per treatment group were required. In addition, this sample size was sufficient to detect as statistically significant a difference of 0.5 log_10_ in the peak of anti-Tat antibodies among the immunization groups, assuming a maximum common standard deviation of 0.8 log_10_, with a significance level of 5% and a power of 80%. Sample size was calculated using nQuery Advisor® software, version 7.0.

Subjects were randomized into 4 treatment groups to ensure unbiased patients allocation as described earlier [22]. The Randomization list was generated by the appointed Contract Research Organization (CRO) using a block size of 4, according to a randomization scheme of 1:1:1:1 (RND PLUS 2.10 software). The randomization assignment was carried out by the CRO via web once eligibility was established by the clinical staff. The vaccine kit number to be assigned to the subject was provided via web within the block’s numbers that the clinical site had received [22].

### Clinical study protocol and amendments

The clinical study protocol “A phase II randomized, open label, immunogenicity and safety trial of the vaccine based on the recombinant biologically active HIV-1 Tat protein in anti-Tat negative HIV-1 infected HAART-treated adult subjects” (dated 12/02/2008), submitted and approved by national competent authorities, included 128 individuals with chronically suppressed plasma viremia (viral load <50 copies/ml in the last 6 months prior to the screening) without a history of virologic rebound, CD4^+^ T cell counts ≥400 cells/μl, pre-HAART CD4^+^ nadir >250 cells/μl (Table 10).

**Table 10:** **Clinical study protocol and amendments**

**Study ** **protocol**	**Date**	**Sample size**	**Follow-up**	**Major inclusion criteria**
Original	12/02/2008	128	48 weeks	Viral load <50 copies/ml in the last 6 months; No history of virologic rebound; CD4^+^ T cell counts ≥400 cells/μl; Pre-HAART CD4^+^ nadir >250 cells/μl No history of AIDS-related opportunistic or neoplastic disease; No concomitant treatment for HBV or HCV infections
First amendment	24/11/2009	160	144 weeks	Viral load <50 copies/ml in the last 6 months; Allowed history of virologic rebound; CD4^+^ T cell counts ≥200 cells/μl; Any pre-HAART CD4^+^ nadir; Allowed history of AIDS-related opportunistic or neoplastic disease; HBV or HCV co-infections were allowed
Second amendment	20/07/2012	160	48 weeks	Same criteria of the first amendment

An interim analysis performed at 48 weeks in 87 subjects whom completed the treatment phase [22] indicated that the Tat vaccine candidate did not raise any safety concern, therefore, after approval by the DSMB, the study was amended (clinical study protocol dated 24/11/2009) to include subjects with less restrictive inclusion/exclusion criteria (history of virologic rebound, CD4^+^ T cell counts ≥200 cells/μl irrespective of pre-HAART CD4^+^ nadir, history of AIDS-related opportunistic or neoplastic disease, HBV or HCV co-infections), and to increase the total number of subjects from 128 to 160, upon recalculation of the sample size. Moreover, the follow-up period of the volunteers was extended for additional two years (from 48 to 144 weeks) to evaluate the persistence of the anti-Tat humoral and cellular immune responses (Table 10).

A further amendment (dated 20/07/2012) was submitted and approved by the Ethics Committees to close the study at 48 weeks of follow-up (Table 10). This was required to initiate an “ad hoc” observational study of follow-up, which was further extended until week 180 from the first immunization given the promising results on disease biomarkers, and to render the study more cost-effective. Volunteers were therefore invited to enter the ISS T-002 Extended Follow-up study (ClinicalTrials.gov NCT02118168), which is currently ongoing.

The interim analysis published previously [22] referred to the pre-amendments clinical study protocol, while the present manuscript reports the 48-week data from the post-amendment, completed trial and the results from a subgroup of study participants for whom a longer follow-up was available (n = 76 up to 96 weeks and n = 45 up to 144 weeks).

### Measurements of anti-Tat antibodies and cellular responses

Anti-Tat Abs were assessed by ELISA as described [22,40,81]. Ab titers equal or higher than 25 for IgM and IgA, or 100 for IgG were considered positive. IFN-γ, IL-2 and IL-4 ELIspot were performed as described [22]. IFN-γ ELIspot was considered positive when spot-forming cells (SFC)/10^6^ cells were ≥30, and fold-increase over control was ≥3. The IL-2 and IL-4 ELIspot were considered positive when fold-increase was ≥3.

T-cell proliferation was assessed by CFSE and expressed as fold-increase, calculated as the ratio of proliferation index with Tat versus controls, and considered positive when ≥2, as described [22].

### Lymphocyte phenotyping

Peripheral blood lymphocytes were phenotyped with the BD Multitest 6-color TBNK reagent with BD Truecount tubes (BD Biosciences, San Jose, CA, USA). Samples were acquired with a FACSCanto flow cytometer (BD Biosciences) and data analyzed with a dedicated software (FACSCanto Clinical Software, BD Biosciences), as described [22].

For naïve, central and effector memory CD4^+^ or CD8^+^ T cell subsets, peripheral blood mononuclear cells were stained with anti-human CD3^+^ (PerCP), CD8^+^ (APC), CD45RA (FITC), CD62L (PE) monoclonal Abs (MultiTEST™ BD Biosciences) and anti-human CD4^+^ (APC-Cy7) Ab (BD Biosciences), acquired by FACSCanto flow cytometer and analyzed with the BD FACSDiva Software (BD Biosciences) as described [22].

T cell subsets were identified by hierarchical gating (morphological, on CD3^+^, and then on CD4^+^ or CD8^+^ T cells). Collective quadrant gates based on CD45RA and CD62L expression on CD3^+^/CD4^+^ or CD3^+^/CD8^+^ T cells identified naïve (CD45RA^+^/CD62L^+^), central memory (Tcm) (CD45RA^−^/CD62L^+^), effector memory (Tem) (CD45RA^−^/CD62L^−^ and CD45RA^+^/CD62L^−^) subsets [22]. Data were expressed as the percentage of CD3^+^/CD4^+^ or CD3^+^/CD8^+^ T cells.

To evaluate the expression of CD38 and HLA-DR on CD8^+^ T cells, whole blood was stained with anti-CD8 FITC/CD38 PE/CD3 PerCP/HLA-DR APC (MultiTEST™ BD Biosciences). Collective quadrant gates based on HLA-DR and CD38 expression on CD8^+^ T cells were established.

### Neutralization of Tat-mediated trimeric Env entry in monocyte-derived dendritic cells by sera

Monocyte-derived DC from healthy donors were cultured and induced to maturation as described [24,32]. Purity of DC was always ≥99%. Tat-mediated Env entry in monocyte-derived DC was assessed as described [32]. Briefly, sera were diluted 1:30 in phosphate buffered saline and incubated for 60 min at 37°C with trimeric Env (0.4 μM) previously premixed for 10 min at 25°C with Tat (0.4 μM) or degassed PBS (control). Samples were then added to DC (2×10^5^ cells/mL) to a 1:5 final dilution and incubated for 10 min at 37°C. Cells were then washed with cold medium and treated for 10 min at 37°C with trypsin-ethylenediaminetetraacetic acid (Life Technologies, Paisley, UK) to remove any externally bound protein. After fixation and permeabilization, DC were stained with rabbit anti-gp120 polyclonal Abs (Chem Progress, Milan, Italy) or purified rabbit IgG control Abs (Sigma-Aldrich, Milan, Italy), followed by FITC-conjugated anti-rabbit Ig (Pierce*,* Rockford, IL, US). Fluorescence was analyzed by flow cytometry, and results expressed as the percentage of Env positive cells as compared to isotype stained samples. A pool of sera from six healthy donors and a monoclonal Ab against Tat (mAb 2A4.1, obtained from the AIDS Research and Reference Reagent Program, Division of AIDS, NIAID, NIH) were used as negative and positive controls, respectively.

### Quantification of HIV-1 DNA by real-time PCR

HIV-1 DNA quantification was performed by SYBR green real-time polymerase chain reaction (PCR), as described [82,83]. DNA was obtained from 1.6 mL of peripheral blood. After incubation for 45 min at 37°C in a lysis buffer (sodium dodecyl sulfate 5%, 8M Urea, 0.3M NaCl, 10 mM Tris–HCl pH 7.5, 10 mM EDTA pH 8.0), DNA was purified by phenol extraction followed by ethanol precipitation and RNase treatment, and amount and quality evaluated with a NanoDrop ND-1000 Spectrophotometer by assessing the ratio of absorbance at 260 nm and 280 nm (NanoDrop Technologies, Wilmington, DE, US). Real-time PCR was performed using the HIV-1 DNA quantitative PCR kit (Diatheva s.r.l., Fano, Italy), on a 7700 Real-Time PCR System (Applied Biosystems, Foster City, CA, US). Each sample was analysed in triplicate. Forward and reverse primers (5’- TAGCAGTGGCGCCCGA -3’ and 5’- TCTCTCTCCTTCTAGCCTCCGC -3’, respectively) were designed to amplify a 161 bp fragment derived from the 5’ long terminal repeat (LTR) U5 end to Gag-Pol start sequence of HIV-1 (GenBank accession no. AF003888). Primers’ specificity for HIV-1 group M has been previously confirmed both in silico using BLAST (http://www.hiv.lanl.gov/content/sequence/HIV/COMPENDIUM/2012compendium.htmL), and by real-time PCR with different HIV-1 subtypes (B, C, F, CRF 01_AE and CRF 02_AG), the reference strains A, D, H, and the complete DNA sequence of HIV-2 ROD (EU Programme EVA Centralised Facility for AIDS Reagents, NIBSC, UK) [83]. The cross-reaction with endogenous retroviral sequences [84] was excluded by testing 150 HIV-1 negative blood donors [83]. HIV-1 DNA copy number was estimated by interpolation of the Ct (threshold cycle), determined on a standard curve with a 10-fold serial dilutions (10^5^ to 10^1^), and 2 copies dilutions of a plasmid containing the 161 bp HIV target region, including the Primer Binding Sites (PBS plasmid), as described [83]. The standard curve was considered valid when the slope was between −3.50 and −3.32 (93-100% efficiency) and the minimum value of the coefficient of correlation (R^2^) was 0.98. The limit of quantification was 2 copies per μg of DNA, with a detection limit of 1 copy and a dynamic range of quantification of 5 orders of magnitude (10^5^ to 10^1^). The reproducibility, assessed by calculating the mean coefficient of variation (CV%) for the Ct values, was determined as 1.4%, confirming quantification in the dynamic range. Results were expressed as log_10_ copies/μg DNA or as log_10_ copies/10^6^ CD4^+^ T cells. In the latter case, it was calculated as the ratio between copies/μg DNA and CD4^*+*^ T cell number present in 1.5× 10^5^ white blood cells (WBC): [(copies/μg DNA)/(CD4^+^/WBC) × 150,000 WBC] × 10^6^.

### Statistical analysis

Two populations were considered for the statistical analyses: the immunogenicity population (155 subjects), representing all randomized individuals who received at least 3 immunizations (excluding also two subjects with poor ARV compliance), and the safety population (168 subjects), representing all randomized subjects who received at least one administration of the vaccine. An “intention-to-treat” analysis was also applied to anti-Tat Abs (n = 168), CD4^+^ T cell number (n = 159) and proviral DNA (n = 150). Since the assessment of lymphocyte subsets and proviral DNA was included in the study second-line laboratory testing, they were performed according to residual specimen availability. Subjects with at least a positive anti-Tat humoral or cellular response at any given time point during the study were defined “responders”. In particular, percentage of subjects producing Anti-Tat Abs (responders) after Tat immunization was referred to patients who developed Abs within 24 weeks of follow-up (time-points: 4, 8, 12, 16, 20, 24 weeks), while the percentage of subjects with cellular responses was referred to patients with at least one positive response within 48 weeks (time-points: 4, 8, 12, 20, 48 weeks).

Ninety-five percent confidence intervals were estimated for the primary endpoints and comparison among treatment groups was performed using the Odds ratio. Cochran-Armitage Trend test was used to evaluate the association between increasing doses (7.5 or 30 μg) and number of administrations (3 or 5 times) with the percentage of subjects producing anti-Tat Abs (responders) after immunization. Kaplan-Meier method was used to assess the cumulative probability of anti-Tat Ab persistence, by treatment groups and compared by the Log-Rank test. Peak intensity of Abs and cellular responses was compared between Tat doses by Wilcoxon rank-sum test. McNemar’s and Wilcoxon signed-rank tests were used to compare pre-post immunization frequencies and intensity of anti-Tat cellular responses, respectively.

Longitudinal analysis for repeated measurements was used to evaluate the changes from baseline of CD4^+^, CD8^+^, B and NK cell number as well as naïve, Tem and Tcm CD4^+^ and CD8^+^ T cell subsets at year 1, 2 or 3, both overall or stratified by drug regimens (NNRTI/NRTI- or PI-based). The same analysis was applied to evaluate changes from baseline of CD38^+^HLA-DR^+^/CD8^+^ T cells and NK cells at year 1, 2 or 3 or by weeks at year 1 stratified by the two 30 μg treatment groups.

Changes from baseline of naïve, Tem and Tcm CD4^+^ or CD8^+^ T cell percentages in OBS subjects were calculated considering as baseline the first visit at which these parameters were evaluated. The same previous analysis was applied to evaluate the changes from baseline of HIV-1 DNA load stratified by treatment groups and antiretroviral regimens. To estimate the decay rate of HIV-1 DNA, a longitudinal analysis was performed using a random-effects regression model with first order kinetic effect; the slope of the decay was determined from a plot of log_10_ DNA (copies/10^6^ CD4^+^ T cells) versus time in weeks [84]. Relationship between the Tat-mediated Env entry in DC and anti-Tat Ab binding titers was assessed by the generalized estimating equations with adjustment for repeated measures in the same patient, after controlling normality assumption of variables distribution (Saphiro-Wilk test). The percentage of DC internalizing Env in the presence or absence of Tat with sera from vaccinees was assessed by the Student’s *t*-test (log_10_ transformation was performed to normalize the data distribution). Neutralizing sera were defined as those capable of inhibiting Env internalization into DC in the presence of Tat by at least 50% as compared to baseline values. The percentage of subjects positive for neutralizing activity was compared between anti-Tat Ab positive and negative individuals by the Fisher's Exact test. Pearson correlation coefficient was used to assess the relationship between Env entry in DC and anti-Tat Ab titers at week 48. Changes from baseline of HIV-1 DNA in subjects with anti-Tat Abs and neutralizing activity were evaluated by analysis of variance for repeated measurements.

No direct statistical comparison was done between vaccinees and OBS subjects, since they belong to two different studies. Statistical analyses were carried out at two-sided with a 0.05 significance level, using SAS® software, version 9.2 (SAS Institute, Cary, NC, US).

### Ethical approval

Studies in humans were conducted according to the principles of the Declaration of Helsinki and all subjects signed an informed consent prior to enrollment. The ISS T-002 clinical trial was approved by the competent authority (General Director of the Coordinator Clinical Center, Policlinico of Modena, Modena) and by the Ethics Committees of each participating clinical center (Policlinico of Modena, Modena; Arcispedale S. Anna, Ferrara; Istituti Fiosterapici Ospitalieri San Gallicano, Rome; Policlinico of Bari, Bari; Ospedale S.M. Goretti Latina; Fondazione S. Raffaele, Milan; Ospedale S. Maria Annunziata Florence; Ospedale Luigi Sacco, Milan; Spedali Civili, Brescia; Ospedale A. di Savoia, Turin).

## Additional file

Additional file 1: Table S1. ISS T-002: adverse events certainly, possibly or probably related to immunization up to 48 weeks, classified by MedDRA SOC and preferred term.

## Abbreviations

HIV-1: Human immunodeficiency virus-type 1
HAART: Highly active antiretroviral therapy
Ab: Antibody
PI: Protease inhibitor
NNRTI: Non-nucleoside reverse transcriptase inhibitor
NRTI: Nucleoside/nucleotide reverse transcriptase inhibitor
DC: Dendritic cells
IFN-γ: Gamma interferon
IL: interleukin
AE: Adverse events
DSMB: Data safety monitoring board
NK: Natural killer
Tcm: Central memory T cells
Tem: Effector memory T cells
ICH: International conference on harmonization of technical requirements for registration of pharmaceuticals for human use
SOC: System organ class
MedDRA: Medical dictionary for regulatory activities
CRO: Contract research organization
HBV: Hepatitis B virus
HCV: Hepatitis C virus
ELISA: Enzyme-linked immunosorbent assay
ELIspot: Enzyme-linked ImmunoSpot
CFSE: Carboxyfluorescein succinimidyl ester
PCR: Polymerase chain reaction
NAC: National AIDS center
