# Supplementary material for: HIV-1 Tat immunization restores immune homeostasis and attacks the HAART-resistant blood HIV DNA: results of a randomized phase II exploratory clinical trial
Source: Retrovirology. 2015 Apr 29;12:33. doi: 10.1186/s12977-015-0151-y (PMC4414440; doi:10.1186/s12977-015-0151-y)
Supplement: Additional file 1: Table S1. — ISS T-002: adverse events certainly, possibly or probably related to immunization up to 48 weeks, classified by MedDRA SOC and preferred term. [file 12977_2015_151_MOESM1_ESM.docx]

**Table S1.** **ISS T-002: adverse events certainly, possibly or probably related to immunization up to 48 weeks, classified by MedDRA SOC and preferred term.**

| **System Organ Class** | **Preferred term** |  | **Tat 7.5 µg, 3x** | **Tat 7.5 µg, 5x** | **Tat 30 µg, 3x** | **Tat 30 µg, 5x** | **Total** |
| --- | --- | --- | --- | --- | --- | --- | --- |
| Blood & Lymphatic System Disorders | Lymphocytic Infiltration | N | 1 |  |  |  | 1 |
|  |  | % | 100.0 |  |  |  | 100.0 |
|  | Total | N | 1 |  |  |  | 1 |
|  |  | % | 100.0 |  |  |  | 100.0 |
| Cardiac Disorders | Bradycardia | N |  | 1 |  |  | 1 |
|  |  | % |  | 50.0 |  |  | 50.0 |
|  | Chest Discomfort | N |  | 1 |  |  | 1 |
|  |  | % |  | 50.0 |  |  | 50.0 |
|  | Total | N |  | 2 |  |  | 2 |
|  |  | % |  | 100.0 |  |  | 100.0 |
| Eye Disorders | Conjunctivitis | N |  | 1 |  |  | 1 |
|  |  | % |  | 33.3 |  |  | 33.3 |
|  | Photophobia | N |  | 1 |  |  | 1 |
|  |  | % |  | 33.3 |  |  | 33.3 |
|  | Visual Impairment | N |  | 1 |  |  | 1 |
|  |  | % |  | 33.3 |  |  | 33.3 |
|  | Total | N |  | 3 |  |  | 3 |
|  |  | % |  | 100.0 |  |  | 100.0 |
| Gastrointestinal Disorders | Nausea | N |  |  |  | 1 | 1 |
|  |  | % |  |  |  | 100.0 | 100.0 |
|  | Total | N |  |  |  | 1 | 1 |
|  |  | % |  |  |  | 100.0 | 100.0 |
| General Disorders & Administration Site Conditions | Asthenia | N | 1 | 0 | 2 | 0 | 3 |
|  |  | % | 14.3 | 0.0 | 22.2 | 0.0 | 7.7 |
|  | Fatigue | N | 0 | 1 | 0 | 0 | 1 |
|  |  | % | 0.0 | 8.3 | 0.0 | 0.0 | 2.6 |
|  | Hyperidrosis | N | 0 | 1 | 0 | 0 | 1 |
| General Disorders & Administration Site Conditions |  | % | 0.0 | 8.3 | 0.0 | 0.0 | 2.6 |
|  | Injection Site Discomfort | N | 0 | 0 | 0 | 1 | 1 |
|  |  | % | 0.0 | 0.0 | 0.0 | 9.1 | 2.6 |
|  | Injection Site Erythema | N | 1 | 2 | 0 | 2 | 5 |
|  |  | % | 14.3 | 16.7 | 0.0 | 18.2 | 12.8 |
|  | Injection Site Irritation | N | 2 | 1 | 1 | 1 | 5 |
|  |  | % | 28.6 | 8.3 | 11.1 | 9.1 | 12.8 |
|  | Injection Site Pain | N | 3 | 5 | 5 | 6 | 19 |
|  |  | % | 42.9 | 41.7 | 55.6 | 54.5 | 48.7 |
|  | Injection Site Swelling | N | 0 | 1 | 0 | 0 | 1 |
|  |  | % | 0.0 | 8.3 | 0.0 | 0.0 | 2.6 |
|  | Malaise | N | 0 | 1 | 0 | 0 | 1 |
|  |  | % | 0.0 | 8.3 | 0.0 | 0.0 | 2.6 |
|  | Pyrexia | N | 0 | 0 | 1 | 1 | 2 |
|  |  | % | 0.0 | 0.0 | 11.1 | 9.1 | 5.1 |
|  | Total | N | 7 | 12 | 9 | 11 | 39 |
|  |  | % | 100.0 | 100.0 | 100.0 | 100.0 | 100.0 |
| Musculoskeletal & Connective Tissue Disorders | Muscular Weakness | N | 0 |  | 1 |  | 1 |
|  |  | % | 0.0 |  | 100.0 |  | 50.0 |
|  | Myalgia | N | 1 |  | 0 |  | 1 |
|  |  | % | 100.0 |  | 0.0 |  | 50.0 |
|  | Total | N | 1 |  | 1 |  | 2 |
|  |  | % | 100.0 |  | 100.0 |  | 100.0 |
| Nervous System Disorders | Agitation | N | 0 | 1 | 0 | 0 | 1 |
|  |  | % | 0.0 | 11.1 | 0.0 | 0.0 | 6.7 |
|  | Aphasia | N | 0 | 1 | 0 | 0 | 1 |
|  |  | % | 0.0 | 11.1 | 0.0 | 0.0 | 6.7 |
| Nervous System Disorders | Disturbance In Attention | N | 0 | 1 | 0 | 0 | 1 |
|  |  | % | 0.0 | 11.1 | 0.0 | 0.0 | 6.7 |
|  | Dysarthria | N | 0 | 1 | 0 | 0 | 1 |
|  |  | % | 0.0 | 11.1 | 0.0 | 0.0 | 6.7 |
|  | Facial Paresis | N | 0 | 1 | 0 | 0 | 1 |
|  |  | % | 0.0 | 11.1 | 0.0 | 0.0 | 6.7 |
|  | Headache | N | 1 | 1 | 0 | 1 | 3 |
|  |  | % | 100.0 | 11.1 | 0.0 | 25.0 | 20.0 |
|  | Paraesthesia Oral | N | 0 | 2 | 0 | 1 | 3 |
|  |  | % | 0.0 | 22.2 | 0.0 | 25.0 | 20.0 |
|  | Presyncope | N | 0 | 1 | 0 | 0 | 1 |
|  |  | % | 0 | 11.1 | 0.0 | 0.0 | 6.7 |
|  | Sciatica | N | 0 | 0 | 0 | 1 | 1 |
|  |  | % | 0.0 | 0.0 | 0.0 | 25.0 | 6.7 |
|  | VII Nerve Paralysis | N | 0 | 0 | 1 | 0 | 1 |
| Nervous System Disorders |  | % | 0.0 | 0.0 | 100.0 | 0.0 | 6.7 |
|  | VI Nerve Paralysis | N | 0 | 0 | 0 | 1 | 1 |
|  |  | % | 0.0 | 0.0 | 0.0 | 25.0 | 6.7 |
|  | Total | N | 1 | 9 | 1 | 4 | 15 |
|  |  | % | 100.0 | 100.0 | 100.0 | 100.0 | 100.0 |
